# KDM6B promotes ESCC cell proliferation and metastasis by facilitating C/EBP*β* transcription

**DOI:** 10.1186/s12885-021-08282-w

**Published:** 2021-05-17

**Authors:** Mei Qin, Fei Han, Jian Wu, Feng-xia Gao, Yuan Li, De-xin Yan, Xue-mei He, Yang Long, Xiao-ping Tang, De-lian Ren, Yan Gao, Tian-yang Dai

**Affiliations:** 1 Department of Immunology, Basic Medicine College, South West Medical University, Luzhou, Sichuan China; 2 Department of Thoracic Surgery, The Affiliated Hospital of Southwest, Medical University, Sichuan Luzhou, China; 3 Experimental Medicine Center, The Affiliated Hospital of Southwest Medical University, Luzhou, Sichuan China

**Keywords:** ESCC, KDM6B, H3K27me3, C/EBP*β*

## Abstract

**Background:**

As an H3K27me3 demethylase and counteracts polycomb-mediated transcription repression, KDM6B has been implicated in the development and malignant progression in various types of cancers. However, its potential roles in esophageal squamous cell carcinoma (ESCC) have not been explored.

**Methods:**

The expression of KDM6B in human ESCC tissues and cell lines was examined using RT-qPCR, immunohistochemical staining and immunoblotting. The effects of KDM6B on the proliferation and metastasis of ESCC were examined using in vitro and in vivo functional tests. RNA-seq and ChIP-seq assay were used to demonstrate the molecular biological mechanism of KDM6B in ESCC.

**Results:**

We show that the expression level of KDM6B increased significantly in patients with lymph node metastasis. Furthermore, we confirmed that KDM6B knockdown reduces proliferation and metastasis of ESCC cells, while KDM6B overexpression has the opposite effects. Mechanistically, KDM6B regulates TNFA_SIGNALING_VIA_NFκB signalling pathways, and H3K27me3 binds to the promoter region of C/EBP*β*, leading to the promotion of C/EBP*β* transcription. Besides, we show that GSK-J4, a chemical inhibitor of KDM6B, markedly inhibits proliferation and metastasis of ESCC cells.

**Conclusions:**

The present study demonstrated that KDM6B promotes ESCC progression by increasing the transcriptional activity of C/EBP*β* depending on its H3K27 demethylase activity.

**Supplementary Information:**

The online version contains supplementary material available at 10.1186/s12885-021-08282-w.

## Background

Esophageal cancer (EC) is one of the most common gastrointestinal malignancies in the world. According to reports in 2018, this disease is ranked as the seventh most frequently diagnosed cancer and the sixth cause of cancer death [1]. In China, esophageal squamous cell carcinoma (ESCC) is currently the major histologic subtype of esophageal cancer [2]. Despite advances in therapeutic methods, ESCC remains one of the most common malignancies in China with an overall five-year survival rate of less than 20% after surgery [3]. Therefore, a better understanding of the molecular mechanisms underlying ESCC progression will supply an arm for improving the diagnosis and treatment of human ESCC.

Epigenetic modification plays an important role in tumorigenesis and development.

Many reports have confirmed that epigenetic modification plays a crucial role in cancer development. As a major epigenetic mechanisms, histone methylation enables the gene promoter to be accessible or inaccessible to transcription factors, thereby regulating target gene expression [4, 5]. Methylation of lysine residue 27 of histone H3 (H3K27), is closely linked to transcriptional repression [6]. Among human cancers, H3K27me3 has been evaluated as a prognostic factor for prostate cancer, breast cancer, ovarian cancer, pancreatic cancer and esophageal cancer [7–9]. H3K27 methylation and demethylation is a dynamic process that can be catalyzed by Methyltransferase (e.g., EZH2), demethyltransferase (e.g., KDM6A and KDM6B), KDM6B also known as JMJD3 [10]. As an important histone demethylase, KDM6B can catalyze the demethylation of H3K27me2/3 to activate target genes by using Fe2+ and α-ketoglutarate as cofactors [11–13]. Besides, KDM6B also associates with transcriptional co-activators to facilitate the transcriptional elongation across the H3K27me3-marked gene body in an enzyme activity-independent manner [14–16]. KDM6B not only contributes to inflammation, but also contributes to various biological processes including development and differentiation [17–20]. Lately, some studies have also shown that KDM6B is implicated in the pathogenesis of several cancers in a context-dependent fashion [21–25]; however, to date, the biological function of KDM6B in ESCC remains to be determined.

Here we investigate the biological impact of KDM6B in ESCC. We show that KDM6B is overexpressed in ESCC patients with lymph node metastasis and confirm that KDM6B can promote ESCC cell proliferation, clone formation, G1/S transformation, migration and invasion. Importantly, we demonstrate that KDM6B, in a demethylase-independent fashion, upregulates C/EBP*β* mediating ESCC cell proliferation and metastasis. Our data, therefore, delineate the biologic function of KDM6B and validate KDM6B may be a novel therapeutic target for ESCC in the future.

## Materials and methods

### Cell lines culture

The human esophageal cancer cell lines (Eca109, Eca9706, TE-1, TE-10, TE-11, KYSE140, and KYSE150) were obtained from the Type Culture Collection of Chinese Academy of Sciences (Shanghai, China) and cultured in RPMI-1640 medium (HyClone, USA) supplemented with 10% Fetal Bovine Serum (FBS) (Gibco-BRL), 100 μg/ml streptomycin and 100 units/ml penicillin G (Beyotime Biotechnology, Shanghai, China) in 5% CO_2_ and humidified atmosphere at 37 °C. Cells at the third generation of the exponentially growing state were used for all experiments.

### IHC analysis in ESCC and adjacent tissues

Samples were collected from 41 patients with ESCC, who underwent complete surgical resection at the Department of Cardiothoracic Surgery of the Affiliated Hospital of Southwest Medical University from January 2017 to March 2018. The clinicopathological features of the ESCC patients are summarized in Table 1. Tissue sections were incubated with rabbit anti-human KDM6B antibody (1100, ab38113, Abcam, Cambridge, UK), IHC scoring was blindly assessed by two Pathology doctor. The immunohistochemistry procedure and scoring.

**Table 1:** clinicopathological characteristics of patient samples and expressionof KDM6B in ESCC

characteristics	Number of cases (%)
Age(y)	
≥ 60	3 (7.3)
< 60	38 (92.7)
Gender	
Male	11 (26.8)
Female	30 (93.2)
T classification	
T1	1 (2.4)
T2	13 (31.7)
T3	23 (56.1)
T4	4 (9.8)
N classification	
N0	23 (56.1)
N1	13 (31.7)
N2	4 (49.8)
Lymph node Metastasis	
No	22 (53.7)
Yes	19 (46.3)
Pathologic differentiation	
Low	6 (14.6)
Medium	18 (43.9)
High	17 (41.5)
Expression of KDM6B	
Low expression	26 (63.4)
High expression	15 (36.6)

of KDM6B expression were performed as previously described [26]. Samples with IHC score < 3 were defined as low expression, while samples with IHC score ≥ 3 were defined as high expression.

### Western blotting

Cells were lysed in Lysis buffer (Beyotime Biotechnology, Shanghai, China) and centrifuged at 4 °C for 15 min. Protein concentrations were measured through BCA Protein Quantitation Assay (Beyotime Biotechnology, Shanghai, China). Total cell lysates were equally loaded on 8–15% SDS-polyacrylamide gel for running and then transferred to polyvinylidene difluoride (PVDF) membranes (Amersham, Buckinghamshire, UK). After blocking for 1 h at room temperature with 5% non-fat milk in Phosphate buffered saline with Tween 20 (PBST), the membranes were incubated for overnight with the primary antibodies anti-KDM6B (abs103127, Absin, 1:1000), anti-Tri-Methyl-Histone H3(Lys27) (C36B11) Rabbit mAb (#9733, Cell Signaling, 1:1000), anti- GAPDH (AG019, Beyotime Biotechnology, 1:5000), anti- Histone H3 Mouse Monoclonal Antibody (AF0009, Beyotime Biotechnology, 1:1000). After staining with corresponding horseradish peroxidase (HRP)-linked secondary antibodies, signal detection was performed using a chemiluminescence phototype-HRP Kit (Beyotime Biotechnology, Shanghai, China) and exposed to film in a dark room.

### RT-qPCR

Total RNA from esophageal tissues was extracted with TRIzol (Invitrogen, Carlsbad, CA) and cDNA was synthesized from 1 μg of total RNA using the ReverTra Ace qPCR RT Master Mix (TOYOBO, Japan) following the manufacturers’ instruction. The mRNA levels of target genes and the internal standard glyceraldehyde 3-phosphate dehydrogenase (GAPDH) were measured by RT-qPCR in triplicate using the QuantiNova SYBR Green PCR kit (Qiagen, Inc.) and the StepOne™ real-time PCR System (Applied Biosystems; Thermo Fisher Scientific, Inc.).The specific primers for the genes are listed in Table 2.

**Table 2:** the specific primers for the genes

Primer Name	Sequence (5′ to 3′)
KDM6B	Forward:TCCAATGAGACAGGGCACAC
	Reverse:CTTTCACAGCCAATTCCGGC
CLDN16	Forward:CTGGGTCTCTGGGTTGCTTT
	Reverse:TTTCTCTCAGGTCCAACATCTTT
MMP28	Forward:AGAGCGTTTCAGTGGGTGTC
	Reverse:AAAGCGTTTCTTACGCCTCA
CXCL2	Forward:CGCCCAAACCGAAGTCATAG
	Reverse:AGACAAGCTTTCTGCCCATTCT
BCL6	Forward:CGGAAGATGAGAGATTGCCCTGC
	Reverse:GCCTGGAGGATGCAGGCATT
JUNB	Forward:CAGGCTCACGTAGCCGTACT
	Reverse:GCTCGGTTTCAGGAGTTTGTAGT
SOX2	Forward:GGCGCAAGATGGCCCAGGAGAAC
	Reverse:CGCCGGGCAGCGTGTACTTATCC
STAT3	Forward:GGAGGAGTTGCAGCAAAAAG
	Reverse:TGTGTTTGTGCCCAGAATGT
STAT5A	Forward:GAAGCTGAACGTGCACATGAATC
	Reverse:GTAGGGACAGAGTCTTCACCTGG
C/EBPβ	Forward:ACGACTTCCTCTCCGACCTC
	Reverse:CAGGCTCACGTAGCCGTACT
GAPDH	Forward:CCACTCCTCCACCTTTG
	Reverse:CACCACCCTGTTGCTGT

### RNA-seq

Total RNA of each sample was extracted using TRIzol Reagent (Invitrogen). Total RNA of each sample was quantified and qualified by Agilent 2100 Bioanalyzer (Agilent Technologies, Palo Alto, CA, USA), NanoDrop (Thermo Fisher Scientific Inc.), and 1% agarose gel. We used 1 μg of total and polysome-associated RNA fractions for RNA-seq from two independent biological sample replicates. The RNA-seq was performed in an Illumina HiSeq platform using RNA-seq kit according to the manufacturer’s recommendation (Illumina, Inc.).The sequences were processed and analyzed by GENEWIZ.

### Establishment of KDM6B knockdown cell line

Gene-specific shRNA sequences were obtained from the Sigma website (http://www.sigmaaldrich.com/), and the hairpin sequences were cloned into the lentiviral vector pLKO-Tet-On with Puromycinas as a selection marker, shRNAs targeting KDM6B were screened.

Lentiviral particles were packaged in HEK 293 T cells by transient transfection. Briefly, 10 μg of shRNA encoding lentiviral plasmid, 5 μg of helper pMD2.G plasmid and 10 μg of helper psPAX2 plasmid were mixed with 30 μl PEI transfection reagent (Sigma-Aldrich). And co-transfected into HEK 293 T cells at 70–80% confluency in a 10 cm Petri dish according to the manufacturer’s protocol. At 72 h post-transfection, the supernatant containing virus particles was collected and filtered through a 0.45 μm PVDF Millex syringe filter.

Each cell line was subjected to a puromycin tolerance test to determine the optimal concentration for establishing stable cell lines. To generate an shRNA-expressing stable cell line, cells were infected with lentiviral particles with 4 μg/ml polybrene. After 48 h, the cells were selected by 1 μg/ml puromycin (GIBCO) for 5 days. And the knockdown efficiency was tested by WB assay and RT-qPCR.

### Plasmid constructs, siRNAs and transfection

To overexpress KDM6B, ESCC cells were transfected with a plasmid containing the full-length cDNA of the human KDM6B gene, which was obtained from Addgene (plasmid ID #24167). The transfections were performed using LipoFiter™ Liposomal Transfection reagent according to the manufacturer’s instructions. The cells were plated in 6 cm dishes and incubated overnight. When the confluence reached 70–80%, the supernatant of the cell was replaced with fresh RPMI-1640 medium supplemented with 10% FBS, and then transfected with 5 μg of either KDM6B-vector using 16 μl LipoFiter™. The media was replaced 6 h after transfection, and the cells were incubated at 37 °C for 48 h. The overexpress efficiencies were confirmed by western blotting and RT-qPCR assay. The indicated siRNAs were synthesized by Guangzhou Riboio Co., Ltd. (China). Cell transfection was performed using *riboFECT*™*CP* (Ruibo Biotechnology Co., Ltd.) according to the manufacturer’s instructions. Cells were seeded in free-antibiotics media in six-well plates and cultured for 24 h before siRNA transfection. Non-targeting control, siRNA groups were set up when the density of cells reached 50%. Approximately 5 μL of 20 μM siRNA was diluted with 120 μL of 1× riboFECT™ CP buffer and added with 12 μL of riboFECT™ CP reagent. The mixture was incubated for 15 min at room temperature. The prepared riboFECT™ CP mixture was added to 1863 μL of cell culture medium. The culture plate was placed in a CO_2_ incubator at 37 °C for 48 h, and the cells were then collected. The efficacy of siRNA transfection was evaluated by western blot and real-time quantitative RT–PCR. The siRNA sequences used in this study are as follows:siRNAs against C/EBP*β* were siRNA-1:5′-GUAUAUUUUGGGAAUCUUUTT-3′,siRNA-2:GUCUAUGUGUACAGAUGAAUG and non-targeting control siRNA, 5′-UAGCGACUAAACACAUCAA-3′.For rescue experiments, plasmids were transfected on day 1 followed by siRNA transfection on day 2 and observation at 48 h after siRNA transfection.

### In vitro colony-formation assays

Cells transfected with the indicated vectors were plated at low density (500 cells per 6-cm plate), incubated in a 5% CO_2_ incubator at 37 °C. The culture medium needed replacement 3 d/time, and the culture was stopped when the clone was visible to the naked eye. With the culture medium discarded, the cells were washed two times with PBS, and Cell clones were immobilized by methanol for 15 min and stained with crystal violet solution (Beyotime Biotechnology, Shanghai, China) for 3 min before photographed and counted. Colonies containing more than 50 cells were counted using a microscope.

### CCK-8 assay

Cell viability was evaluated using the Cell counting kit-8 (CCK-8) assay. The cells were detached and inoculated into 96-well plates at a density of 1000 cells/well (200 μL in each well). Four wells with cells were set for each group, and blank and control wells were also set. The plate was incubated in a 5% CO_2_ incubator at 37 °C for 96 h. After the cells adhered to the wall, With the culture medium discarded, fresh culture medium containing 10 μL CCK-8 reagent (Beyotime Biotechnology, Shanghai, China) was added for incubation for 2 h. Subsequently, an enzymatic marker (Bio-Rad, USA) was used to detect the OD value at the wavelength of 450 nm. The cell survival rate was calculated, and the cell growth curve was drawn. The experiment was repeated three times.

### Wound scratch based cell migration assay

In the scratch based wound healing assay [27] cells were seeded in a 12-well plate and cultured until confluence. Then cells were serum-starved for 12 h before scratching. Wound scratching was performed using 200 μl pipette tips, and cells were gently washed twice with PBS. Pictures were taken immediately. Finally, cells were treated with RPMI-1640 medium with 2% FBS. The wound healing rate was measured by a comparison of the images using the closure distance of cells after 0 h and 24 h.

### Cell migration assay

A migration assay was performed using Transwell inserts (Corning-Costar, Cambridge, MA) that contained a polycarbonate transwell membrane filter (6.5 mm diameter, 8 μm pore size). Cells were maintained at a concentration of 5 × 10^5^ cells/ml in serum-free RPMI-1640. A total of 200 μl cell suspension was added into the upper chamber, whilst the lower chamber was treated with 500 μl of RPMI-1640 with 10% FBS at 37 °C for 24 h. The medium was discarded, and non-migrating cells on the top surface of the upper chamber were removed gently using cotton swabs. Migrated cells were fixed with pre-chilled methanol for 30 min then stained with 0.5% crystal violet at room temperature for 20 min. Representative images were taken under an inverted microscope (magnification, × 10, and × 20) equipped with a camera (Olympus, Japan).

### Cell invasion assay

For the invasion assay, the upper chamber was pre-coated with Matrigel (BD Pharmingen). Ice-cold Matrigel was mixed with ice-cold RPMI-1640 medium at a ratio of 1:8 and spread onto the upper chamber (100 μl/chamber), which was subsequently incubated at 37 °C for 2 h. The following steps, including cell plating, incubation, fixing, and de-staining, were conducted as aforementioned for the migration assay.

### Xenograft models

A total of 18 male BALB/C nude mice (5 weeks of age, 18–20 g) were raised in laminar shelves without specific pathogen conditions with constant temperature, constant humidity, and regular disinfection. Bedding, drinking water and feed were replaced regularly under aseptic conditions. When the KYSE150 sh-NC and sh-KDM6B-1 cells were at a logarithmic growth phase, they were subcutaneously injected into the armpit of the forelimb of nude mice (0.2 mL, 2 × 10^6^ cells). The tumor length (a) and the short diameter (b) were measured using a calliper twice a week, and the tumor growth curve was drawn via V = ab^2^/2. Three weeks after the injections, the mice were euthanized in a CO2 chamber, followed by cervical dislocation and lack of withdraw reflex to assure animal death. The tumours were removed and photographed.

Tumor metastases were generated by intravenously injecting 2 × 10^5^ KYSE150 sh-NC and sh-KDM6B-1 cells into the tail vein of mice. After 30 days, all mice were euthanized and examined for tumor metastases. Their lungs were removed and fixed in formalin and processed for HE analyses. The methods used in animal experiments were performed following the relevant guidelines.

### GSEA (gene-set enrichment analysis)

GSEA was performed using the Broad Institute web platform by pre-ranking the RNA-seq list based on log2-fold change.

### ChIP (chromatin immunoprecipitation) assay

ChIP assays were performed using Simple ChIP Plus Enzymatic Chromatin IP Kits (CST, #9003) according to the manufacturer’s instructions. Briefly, cells cultured in 15 cm Petri dishes were crosslinked with 1% formaldehyde when reaching 70–80% confluence. Crosslinking was terminated by adding 10 × glycine followed by rinsing the cells twice with cold PBS, and 4 × 107 cells were collected into one 15 ml conical tube. The cell pellet was resuspended and incubated in Buffer A on ice for 10 mins for cell lysis. The nuclei were collected by centrifugation at 3000 rpm for 5 mins at 4 °C and resuspended in Buffer B followed by centrifugation and supernatant removal. The nuclei pellets were resuspended in 1.0 ml Buffer B, and transferred to a 1.5 ml microcentrifuge tube. 7.5 μl of Micrococcal Nuclease was added and incubated for 20 mins at 37 °C to digest the DNA to lengths of approximately 150–300 bp. After the termination of digestion by adding 100 μl of 0.5 M EDTA, the nuclei pellets were collected by centrifuging at 13,000 rpm for 1 min at 4 °C. The nuclear pellets were resuspended in 1 ml ChIP buffer and separated into two tubes, and incubated on ice for 10 mins followed by subsequent sonication of lysates at 9 W, 10 s ON/30 s OFF, nine cycles. Lysates were collected by centrifugation at 10,000 rpm for 10 mins at 4 °C. The supernatant (single nucleosome lysate) was transferred to a new tube, and stored at − 80 °C for the following experiments. 50 μl of lysate was used for the analysis of chromatin digestion.

Pulldown: The single nucleosome lysate was diluted with ChIP buffer with protease inhibitor cocktail (for each pulldown, 200 μl lysate was added into 300 μl 1 × ChIP Buffer). 10 μl of sample was saved as 2% input. To reduce nonspecific binding, the lysate was pre-cleared with 10% BSA and 30 μl ChIP Grade Protein G Magnetic Beads (CST, #9006) at 4 °C with rotation for 1 h. The supernatant was collected by placing the tube on a magnetic separation rack. Antibodies of H3K27me3 or IgG control were added and incubated on rotation at 4 °C overnight followed by incubation with 30 μl of magnetic beads for 2 h at 4 °C. Beads were washed with low salt wash buffer (100 μl 10 × ChIP Buffer in 900 ml water) three times and with high salt wash buffer (100 μl 10 × ChIP Buffer and 70 μl 5 M NaCl in 830 μl water) once on rotation. Then, beads were eluted with 150 μl of ChIP elution buffer for 30 mins at 65 °C with gentle vortexing (1200 rpm) in thermomixer. Eluted supernatant was incubated with 6 μl 5 M NaCl and 2 μl Proteinase K at 65 °C for 2 h in thermomixer to digest the protein. DNA was purified using spin columns, and sent for ChIP-seq or ChIP-PCR analysis. For ChIP-PCR, ll ChIP signals were normalized to the input, and relative fold-change was compared with IgG controls. The primers of C/EBP*β* were: 5′- CATCAAGCAAAACGCTATGGG-3′ and 5′- CTCAGAACCTAGAGCCGGAAA-3′. The IP efficiency was calculated using the equation shown below.

$$ \mathrm{Percent}\ \mathrm{Input}=2\%\times {2}^{\left(C\left[\mathrm{T}\right]\mathrm{Input}\ \mathrm{Sample}-C\left[\mathrm{T}\right]\ \mathrm{IP}\ \mathrm{Sample}\right).} $$

*C* [T] = *C*T = Threshold cycle of PCR. With this method, signals obtained from each immunoprecipitation are expressed as a percent of the total input chromatin.

### ChIP-seq and data analysis

ChIP samples were quantified using a Qubit 2.0 Fluorometer (Invitrogen, Carlsbad, CA, USA) and qualified by Agilent Bioanalyzer 2100(Agilent Technologies, Palo Alto, CA, USA). Next-generation sequencing library preparations were constructed following the manufacturer’s protocol (NEBNext® Ultra™II DNA Library Prep Kit for Illumina®). The sequences were processed and analyzed by GENEWIZ. The ChIP-seq peaks were visualized using IGV (http://www.broadinstitute.org/igv/).

### Statistical analysis

All experiments were performed in three independent experiments (except RNA -seq and ChIP-seq), and data were expressed as mean ± standard error (SE). GraphPad Prism 6.0 (GraphPad Software Inc., La Jolla, CA, USA) was used to analyze the data. ANOVA and Student’s t test were used for significance analysis. *P* < 0.05 was considered to indicate statistical significance. Data were assessed for normality using a Shapiro-Wilks normality test rejecting normality at *p* < 0.05.

## Results

### KDM6B expression was significantly increased in metastatic ESCC

**Fig. 1:**
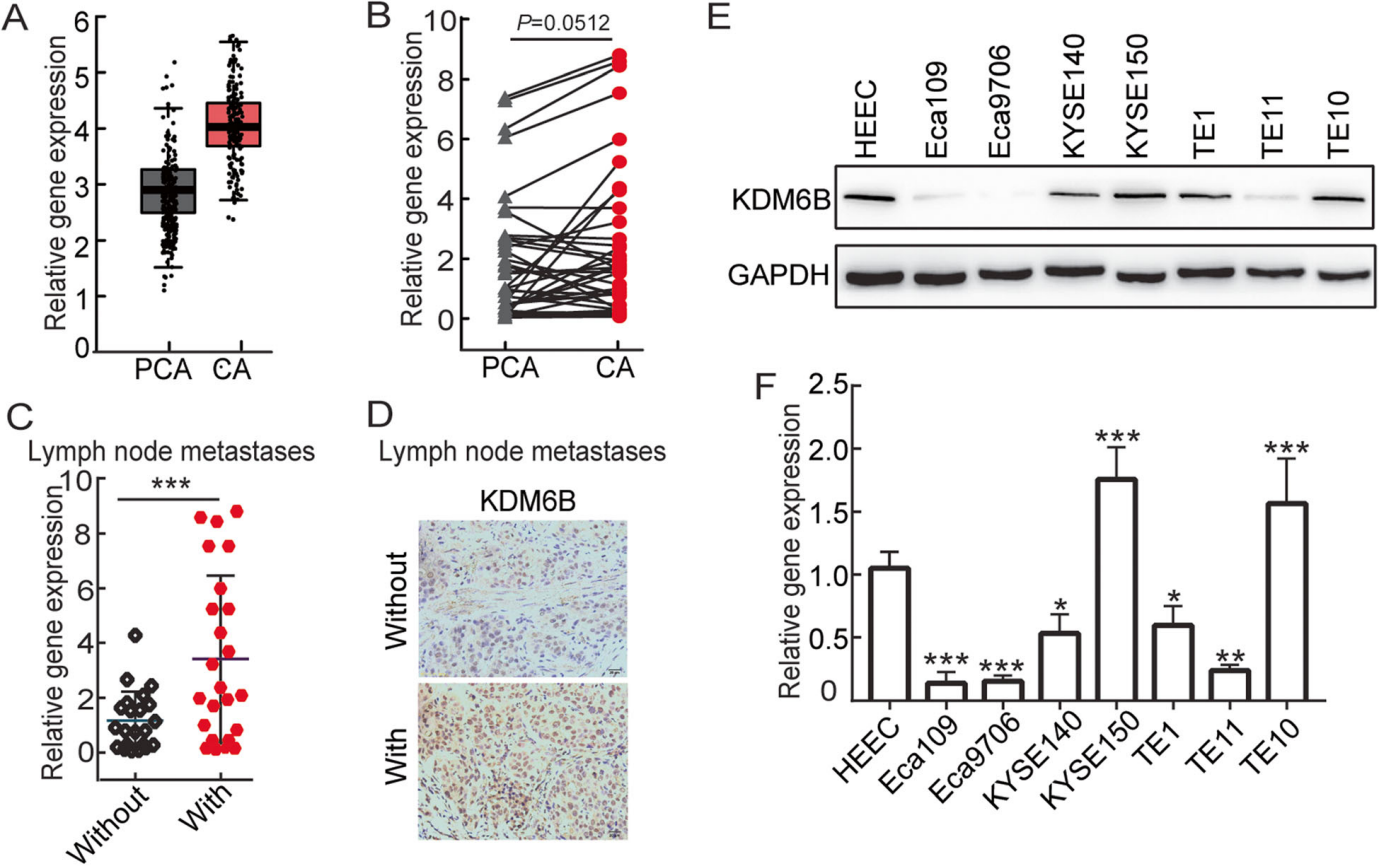
KDM6B expression was significantly increased in metastatic ESCC. **a** TCGA data shows the expression levels of KDM6B in 182 ESCC tissues and 286 normal esophageal tissues. **b** Relative levels of KDM6B in 41 surgical specimens of ESCC and matched adjacent nonmalignant tissues were quantified by quantitative real-time polymerase chain reaction (qRT-PCR). CA, ESCC tissues; PCA, normal esophageal tissues. **c**,**d** Means of KDM6B relative levels from ESCC tissues including a group of 19 ESCC patients with positive lymph node metastases compared with another group of 22 ESCC patients with negative lymph node metastases, and representative IHC images(**d**). **e**, **f** Western blot and RT-qPCR data for KDM6B protein and mRNA expression in Normal esophageal epithelial cells and seven ESCC cell lines. Glyceraldehyde 3-phosphate dehydrogenase (GAPDH) serves as an internal normalized reference for KDM6B expression levels.* *p* < 0.05, ** *p* < 0.01, *** *p* < 0.001. ESCC, esophageal squamous cell carcinoma

TCGA data set showed that there was no significant difference in *KDM6B* mRNA expression between esophageal cancer tissues and normal esophageal tissues, but the overall trend was upregulated (Fig. 1a). To further validate this result, we used qRT–PCR assays to evaluate KDM6B levels in 41 primary ESCC tissues and paired normal esophageal tissues, and KDM6B was not significantly upregulated in the ESCC tissues (Fig. 1b). However, the expression level of KDM6B was obviously increased in patients with lymph node metastasis (Fig. 1c,d). and analysis of the adjacent non-tumor esophageal tissues and ESCC tissues by IHC revealed that KDM6B expression is associated with the lymph node metastasis and the N stage of ESCC, but not with age, sex, T stage or Pathologic differentiation (Table 3). To further study the function of KDM6B in ESCC, we next examined the expression of KDM6B among HEEC cells and ESCC cell lines by Western blot and RT-qPCR (Fig. 1e,f, Sup Fig. 3A). Compared with HEEC, the expression of KEM6B was significantly higher for KYSE150, TE10, and significantly lower for Eca9706, TE11.

**Table 3:** correlation between KDM6B expression and clinicopathologic characteristics of ESCC patients

characteristics KDM6B	KDM6B		
	Low or none, no. cases	High, no. cases	P value
Age(y)			
≥ 60	6	5	0.406
< 60	12	18	
Gender			
Male	16	22	0.409
Female	2	1	
T classification			
T1	1	0	0.233
T2	8	5	
T3	8	15	
T4	1	3	
N classification			
N0	16	7	0.001
N1	1	12	
N2	1	3	
Lymph node Metastasis			
No	16	6	0.000
Yes	2	17	
Pathologic differentiation			
Low	1	5	0.342
Medium	9	9	
High	8	9	

### KDM6B promotes the proliferation of ESCC cells

**Fig. 2:**
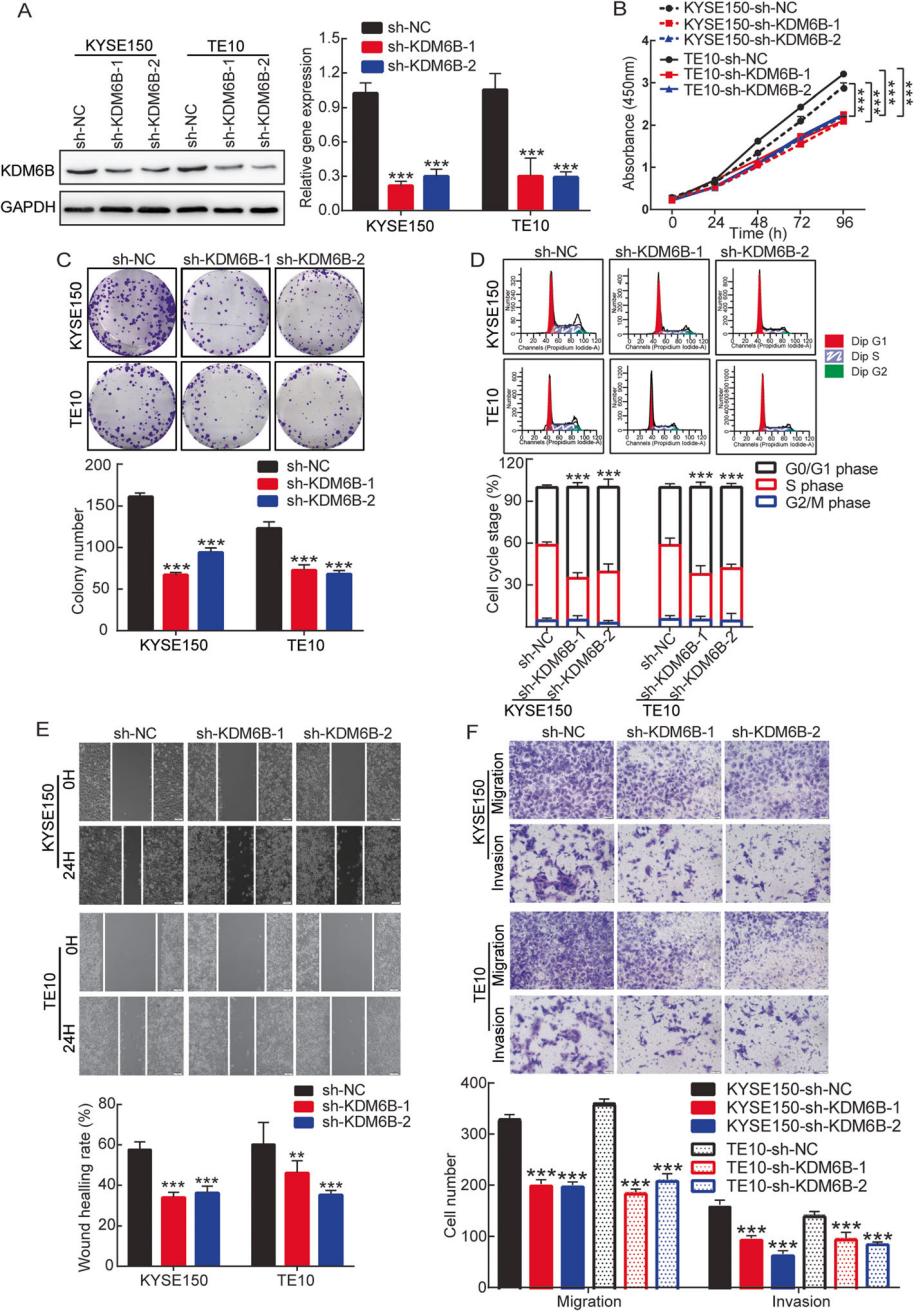
The knockdown of KDM6B KDM6B inhibites the proliferation and Metastasis of ESCC cells. **a** Western blotting (left panels) and RT-qPCR (right panels) were performed to measure the KDM6B protein and mRNA expression level changes following KDM6B interference. **b** The CCK-8 assay was used to determine cell viability after interfering KDM6B every 24 h for 4 days. **c** ESCC cells were seeded at a density of 500 cells/well for clone formation assays. Representative crystal violet staining of clone formation (upper panels) and statistical graphs of clone numbers (lower panels). **d** Cell cycle distribution was analyzed following the silencing of KDM6B in KYSE150 and TE10. Representative images of cell cycle distribution (upper panels) and statistical graphs of cell cycle changes (lower panels). **e** Cell migration was determined using the wound healing migration assay (magnification, × 100) Representative images of KYSE150 and TE10 lentivirus stable transfected at 0 and 24 h of wound healing assay, image j was used to analyze the results of wound scratch images. A bar graph shows the quantitative results of scratch healing. **f** Cell migration or invasion was determined using Transwell migration or invasion assay. Representative images of crystal violet stained for lentivirus stable transfected of KYSE150 and TE10 by transwell migration or invasion assays (upper panels). A statistical graph shows Cell number (lower panels). Results are representative of three independent experiments and are expressed as the mean ± S.D. * *p* < 0.05, ** *p* < 0.01, *** *p* < 0.001

Then, we transfected short hairpin RNA (shRNA) into KDM6B highly expressed cell lines KEYS150 and TE10 to knockdown KDM6B expression. Subsequently, stably transfected cell lines were screened by puromycin for 5 days after 2 days of infection. At the same time, We transfected HA-KDM6B overexpression plasmid for 3 days into KDM6B lowly expressed cell lines Eca9706 and TE11. We use Western blot and RT-PCR to determine the knockdown and overexpress efficiency. Respectively, compared with the sh-NC group, the mRNA and protein expressions of KDM6B were significantly inhibited in cells transfected with sh-KDM6B (Fig. 2a, Sup Fig. 3B); and KDM6B was largely overexpressed in cells transfected with HA-KDM6B (Sup Fig. 1A). The successful establishment of a KDM6B gene silencing lentivirus and overexpressing vector provided a useful tool for investigating the function of KDM6B in ESCC cell lines.

We assessed tumor cell proliferation in response to KDM6B interference or overexpression, using the CCK-8 assay. As shown in Fig. 2b and Sup Fig. 1B, the Interference of KDM6B in KYSE150 and TE10 exhibited significantly reduced proliferation compared with controls at 96 h, and overexpressed KDM6B promoted the proliferation of Eca9706 and TE11 cells. Coincidence with CCK8 assay, the Interference of KDM6B also significantly decreased the clone formation ability of KYSE150 and TE10 cells, and the overexpression of KDM6B in Eca9706 and TE11 promoted the clone formation ability of ESCC cells (Fig. 2c, Sup Fig. 1C). Furthermore, the cell cycle was also analyzed to evaluate the KDM6B silencing or overexpressing effect. The results demonstrated that knockdown of KDM6B markedly increased the proportion of cells in the G1 phase, but decreased the proportion of cells in the S phase in KYSE150 and TE10 cells; the overexpress of KDM6B induced Eca9706 and TE11 transition from G0/G1 to S phase (Fig. 2d, Sup Fig. 1D). These results indicate that KDM6B regulates cell cycle distribution, promotes the proliferation and Clone formation of tumor cells.

### KDM6B promotes migration and invasion of ESCC cells

We also investigate whether the KDM6B has a function as a vital role in the migration and invasion of ESCC cells. Migration and invasion capability were detected using wound healing assay and Transwell migration & invasion assay. Figure 2e and Sup Fig. 1E showed when KYSE150 and TE10 stably infected by Lv-sh-KDM6B, the migration rate significantly decreased which compared with negative control (*p* < 0.05), and Eca9706 and TE11 transiently transfected with HA-KDM6B overexpression plasmid, the migration rate increased which compared with negative control(*p* < 0.05). The Transwell assay also revealed similar results when stable infection by the Lv-sh-KDM6B and transient transfected by the HA-KDM6B overexpression plasmid. Lv-sh-KDM6B interference reduced the KYSE150 and TE10 migration and invading cell number, and HA-KDM6B overexpression increased the Eca9706 and TE11 migration and invading cell number, which compared with the control group (Fig. 2f, Sup Fig. 1F). The above results suggested that KDM6B can promote ESCC cell migration and invasion.

### The interference of KDM6B inhibits ESCC growth and distal pulmonary metastases in vivo

**Fig. 3:**
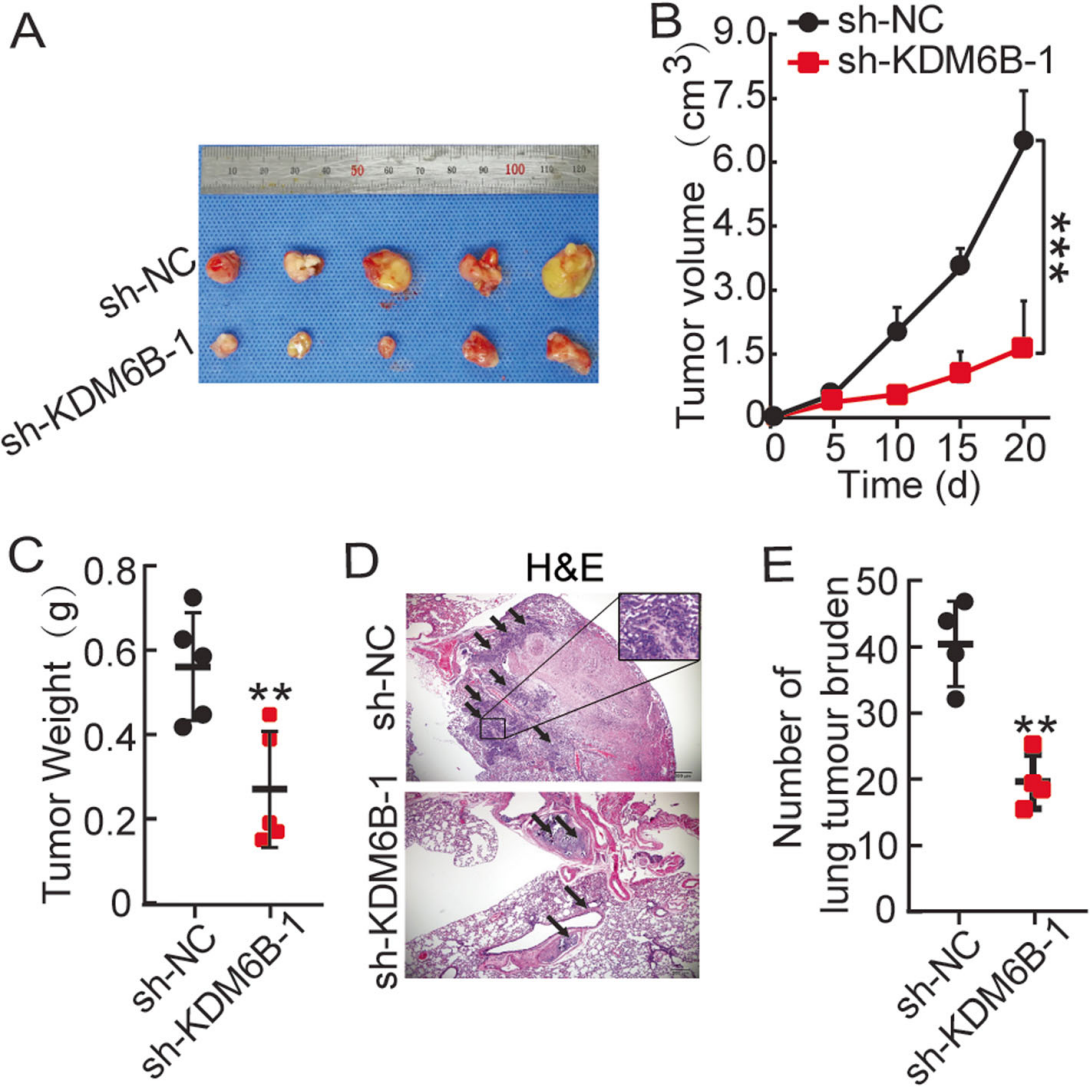
The interference of KDM6B inhibits ESCC growth and distal pulmonary metastases in vivo. **a** Photographs of mice after subcutaneous xenografting injection with ESCC cells stably interfering KDM6B and Lv-control (*n* = 5). **b** Tumor growth curve of each group. **c** Tumor weight averages between control and sh-KDM6B-1 groups at the end of the experiment (Day 21). Data are presented as mean ± SD (n = 5). **d** Photographs of mice in lung tissues after tail veins injection with ESCC cells stably down expressed KDM6B and sh-NC. Hematoxylin and eosin staining of lung tissue isolated from nude mice that had been injected with ESCC cells stably down expressed KDM6B and sh-NC via lateral tail veins (magnification, × 200) (left panels). **e **The calculated number of metastases in mice that had received lateral tail injections. ** *p* < 0.01, *** *p* < 0.001

To investigate the role of KDM6B in vivo, we injected KDM6B-knockdown or control KYSE150 cells subcutaneously into athymic nude mice. We examined tumor volume over time and observed that KDM6B interference significantly repressed tumor growth rates and decreased tumor weight (Fig. 3a,b,c). To further investigate the effect of KDM6B on tumor metastasis, we performed pulmonary metastasis assay in nude mice by injecting sh-KDM6B-1 and sh-NC transfected KYSE150 cells through the tail vein. The result showed that the number of nodules in the sh-KDM6B-1 group was significantly increased when compared to those in the sh-NC group (Fig. 3d,e). These results suggested that knockdown KDM6B could inhibit KYSE150 cell migration and invasion both in vitro and in vivo.

### KDM6B promotes C/EBP*β* transcription via modulating H3K27 methylation

**Fig. 4:**
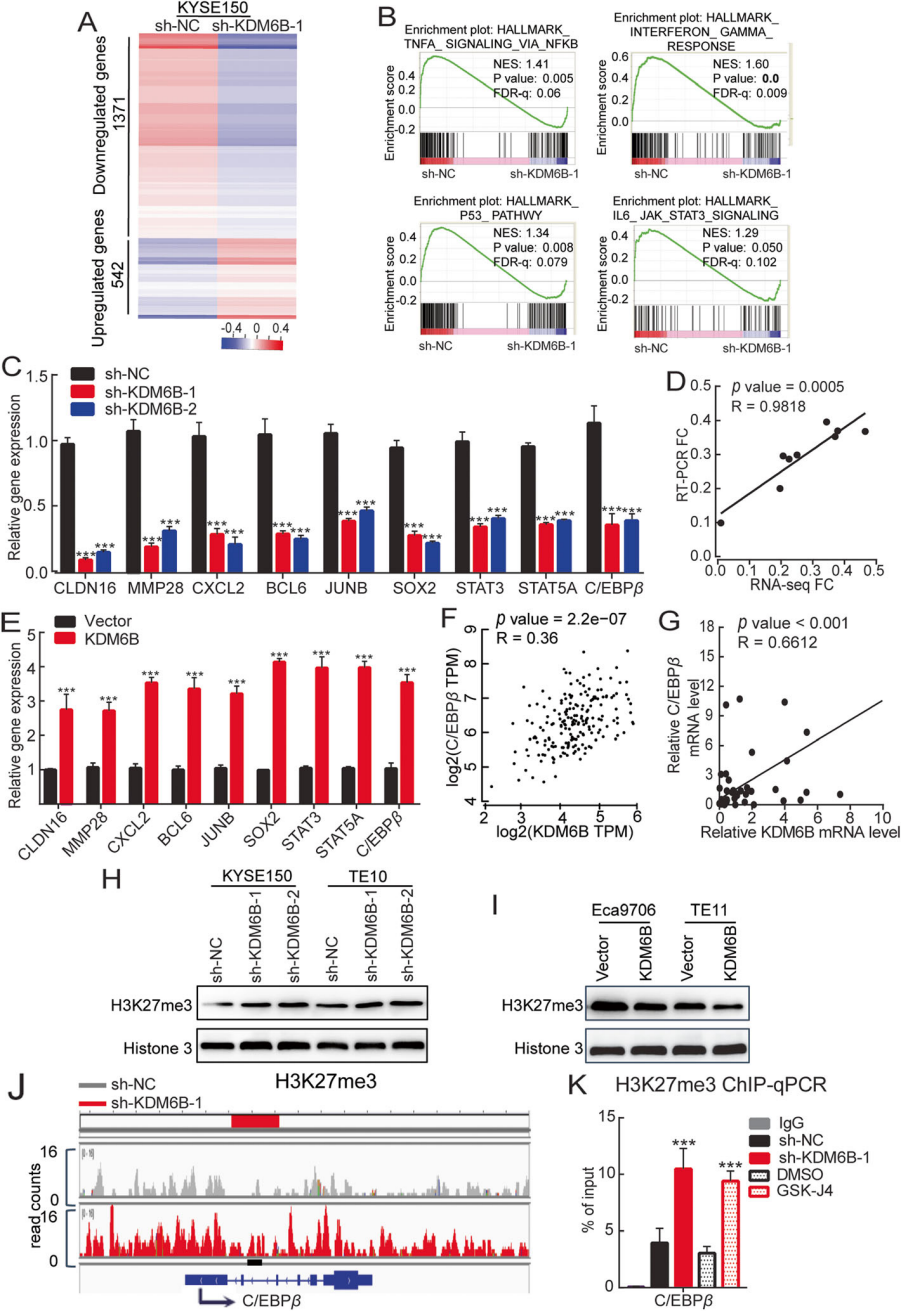
KDM6B promotes C/EBP*β* transcription via modulating H3K27 methylation. **a** RNA-seq results showed the gene expression differentiation between the sh-NC group and the sh-KDM6B-1 group through heat map analysis. Compared with the sh-NC group, the number of down-regulated genes was 1371 and the number of up-regulated genes was 542 in the sh-KDM6B-1 group (fold change≥2,p < 0.05). Red means up, blue means down. The data are representative of 2 biological repeats. **b** GSEA analysis of the RNA-seq data showed the enriched pathway of TNFA_SIGNALING_VIA_NFκB, INTERFERON_ GAMMA_RESPONSE, P53, and IL6_JAK_STAT3_SIGNALING pathway in KYSE150 cell that KDM6B was knockdown as compared with Ctrl cells. ES, enrichment score; FDR, false discovery rate. **c**,**d** RT-qPCR verified the expression of target genes after KDM6B was knocked down in KYSE150. **e** RT-qPCR verified the expression of target genes after over-expressing KDM6B in Eca9706. **f**,**g** Correlation between the expression levels of KDM6B and C/EBP*β*, a key gene of TNFA_SIGNALING_VIA_NFκB signaling pathways in ESCC patient datasets from TCGA(**f**) and our ESCC simple(**g**). All expression data were log (2) transformed. The correlation coefficient (r) and *p* values were detected by “Pearson Correlation”. **h** Western blot was used to detect the expression of H3K27me3 in KYSE150 and TE10 cells after the KDM6B knockdown. **i** Western blot detected the expression of H3K27me3 in Eca9706, TE11 after over-expressing KDM6B for 72 h. **j** Genomic visualization analysis shows a representative snapshot of the H3K27me3 binding site at the C/EBP*β* locus in KYSE150 cells in the control and KDM6B knockdown groups. The long red line is the promoter region, and the short black line is the primer design site. The arrow is the direction of gene transcription. **k** ChIP-qPCR analysis of H3K27me3 ChIP products from the control and shRNA or GSK-J4 treated KYSE150 cells (*n* = 3, values are expressed as mean ± s.d.). * *p* < 0.05, ** *p* < 0.01, *** *p* < 0.001

To investigate the possible regulatory genes, RNA-Seq (RNA sequencing) of KYSE150 cells under KDM6B knockdown using sh-RNA has been performed. RNA-seq results showed that compared with the control group, after knocking down KDM6B, the number of down-regulated genes and up-regulated genes was 1371 and 542, respectively (Fig. 4a). Through gene set enrichment analysis (GSEA), we found that the TNFA_SIGNALING_VIA_NFκB, INTERFERON_ GAMMA_RESPONSE, P53, and IL6_JAK_STAT3_SIGNALING pathway, which were known to be closely associated with oncogenesis, were significantly suppressed in KYSE150 cells that KDM6B was knockdown as compared with Ctrl cells (Fig. 4b). To further verify RNA-Seq’s results, we used RT-qPCR to identify some gene’s expression that were significantly changed in RNA-seq and related to tumor progression, RT-qPCR showed that after interfering with KDM6B, the expression of *CLDN16,MMP28,CXCL2,BCL6, JUNB, STAT3,STAT5A and C/EBPβ* mRNA was lower than that of the control group. Correlation analysis showed that RNA-seq was closely related to RT-qPCR results. In addition, KDM6B overexpression group mRNA expression of these genes is higher than the control group (Fig. 4c,d,e). Importantly, CXCL2,BCL6, JUNB, STAT5A and C/EBP*β* play important roles in the TNFA_SIGNALING_VIA_NFκB pathway. Next we found that *KDM6B* mRNA level was positively correlated with *C/EBPβ* mRNA level among ESCC blast samples in TCGA (Fig. 4f). Moreover, we analyzed the ESCC patient and found a significant positive correlation between the expression levels of KDM6B and C/EBP*β* (Fig. 4g). Our results indicate that the regulation of C/EBP*β* by KDM6B was generally present in ESCC cells.

The above data indicate that C/EBP*β* is a potential KDM6B target gene. However, the mechanism of how KDM6B regulates C/EBP*β* remains to be clarified. KDM6B is an important histone demethylase in cells, mainly through the demethylation of H3K27me3 to promote downstream gene transcription. To further analyze the downstream molecular mechanism of KDM6B in ESCC, we first clarified whether KDM6B exerts demethylase activity in ESCC cells and catalyzes H3K27me3 demethylation. As shown in Fig. 4h,i, Sup Fig. 3C,D, we found that after interfering with KDM6B, the expression of H3K27me3 was enhanced, and after overexpressing of KDM6B, the expression of H3K27me3 was significantly reduced. These results showed that KDM6B exerted a demethylation effect on H3K27me3 in ESCC cells.

Then we performed ChIP-seq and ChIP–qPCR assays to compare the possible alterations H3K27me3 of the KDM6B regulated genes after KDM6B knockout in KYSE150 cells. The KDM6B loss globally increased the H3K27me3 abundance around numerous promoters in KYSE150 cells. As expected, the expression of C/EBP*β* in the KDM6B knockout KYSE150 cells were associated with an increased H3K27me3 around the promoter within 2 kb upstream or 100 bp downstream of the transcription start site (TSS),which is shown in the Integrative Genomics Viewer (IGV) software and validated by ChIP–qPCR (Fig. 4j,k). In parallel, We used GSK-J4 to inhibit KDM6B in ESCC cells, and ChIP-qPCR also found that inhibiting KDM6B can significantly reduce the expression level of H3K27me3 in the *C/EBPβ* promoter region (Fig. 4k). These results indicated that C/EBP*β* in ESCC cells belonged to the direct target genes of KDM6B.

### KDM6B-stimulated proliferation and metastasis of ESCC cells through Upregulation of C/EBP*β* expression

**Fig. 5:**
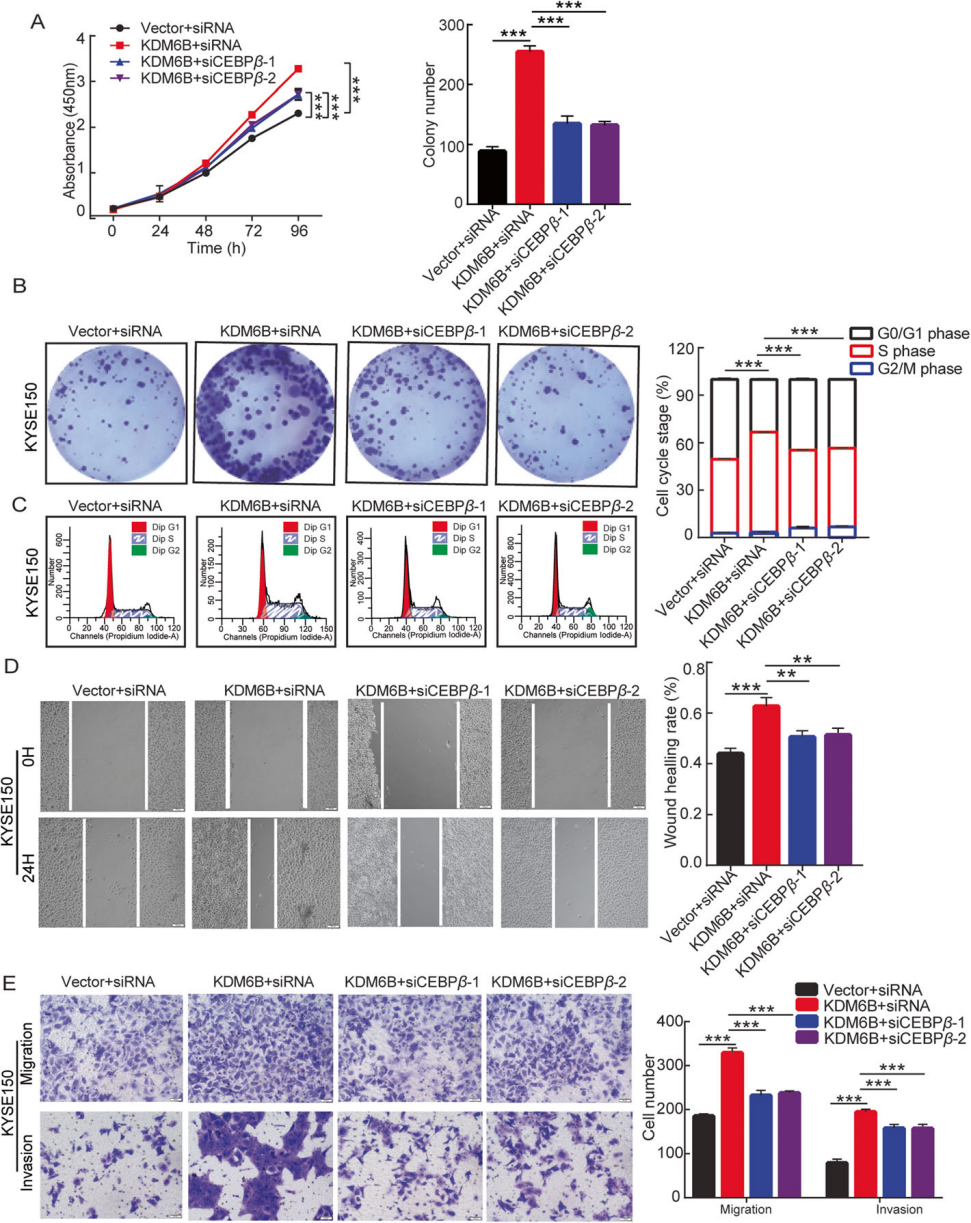
KDM6B-Stimulated proliferation and Metastasis of ESCC Cells through Upregulation of C/EBPβ Expression. KYSE150 cells were successfully cotransfected with KDM6B and C/EBP*β.* **a** The CCK-8 assay was used to determine cell proliferation every 24 h for 4 days. **b** Representative crystal violet staining of clone formation (left panels). and statistical graphs of Clone numbers (upper right panels). **c** Representative images of cell cycle distribution (left panels) and statistical graphs of cell cycle changes (right panels). **d** Cell migration was determined using the wound healing migration assay (magnification, × 100) . Representative images of cells at 0 and 24 h of wound healing assay. Image j was used to analyze the results of wound scratch images. A bar graph shows the quantitative results of scratch healing. **e** Cell migration or invasion was determined using Transwell migration or invasion assay. A representative images of crystal violet stained for KYSE150 by transwell migration or invasion assays. A statistical graph shows cell number (right panels). Results are representative of three independent experiments and are expressed as the mean ± S.D. * *p* < 0.05, ** *p* < 0.01, *** *p* < 0.001

**Fig. 6:**
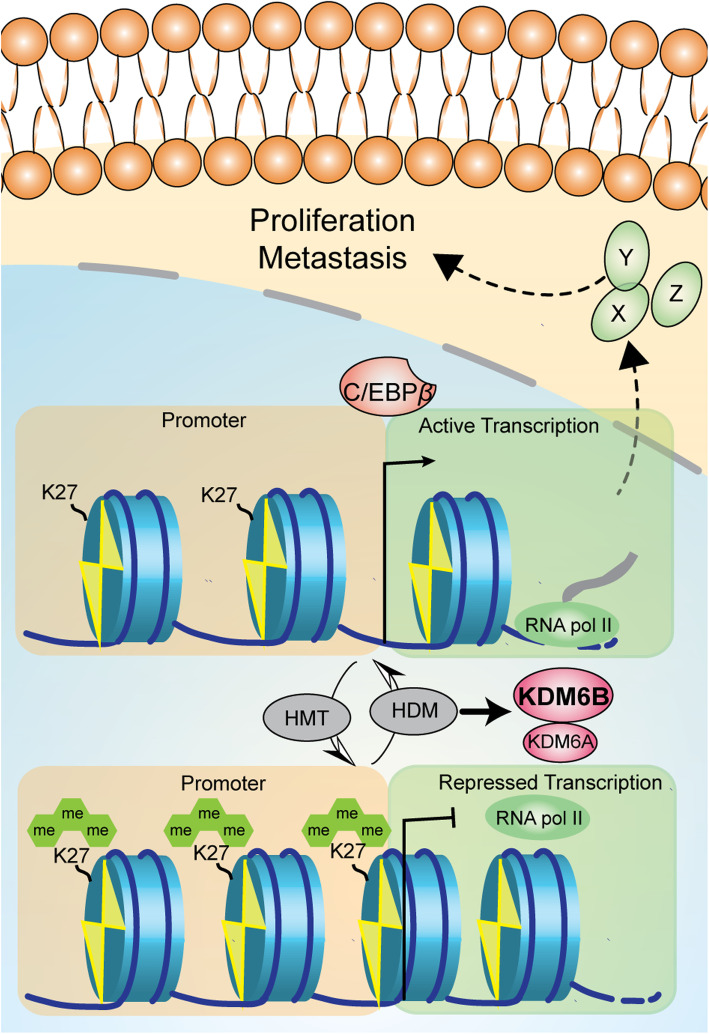
Diagram of the molecular mechanisms underlying KDM6B promotes ESCC progression by increasing the transcriptional activity of C/EBPΒ depending on its H3K27 demethylase activity. Black arrows with dashed lines indicate promotion whereas bar-headed lines represent inhibition

Previous studies have shown that C/EBP*β* was involved in cancer cell growth and tumor progression [28–30], including breast cancer, Pancreatic cancer etc. Our study has revealed that C/EBP*β* promotes proliferation, migration and invasion in ESCC cells (Sup Fig. 2). To investigate whether KDM6B promotes proliferation and metastasis through upregulation of C/EBP*β* expression, C/EBP*β* was knocked down by siRNAs and overexpressed by transfection of KYSE150 cells with HA-KDM6B. Tumor proliferation ability was examined using the CCK8 assay (Fig. 5a), clone formation assay (Fig. 5b) and cell cycle analysis (Fig. 5c). Compared with the control cells, overexpress of KDM6B in ESCC cells resulted in significant promotion of proliferation, clone formation and cell-cycle transition. Importantly, the knockdown of C/EBP*β* rescued proliferation, tumor formation and G1-S conversion of KYSE150 cell overexpressed by KDM6B. Tumor metastasis ability was examined using the wound healing (Fig. 5d) and Transwell assays (Fig. 5e). Compared with the control cells, overexpression of KDM6B significantly promoted the metastasis of KYSE150 cells. Furthermore, compared with only upregulation of KDM6B, concomitant overexpression of KDM6B and downregulation of C/EBP*β* significantly decreased the metastatic ability of KYSE150 cells. In summary, KDM6B is recruited to the chromatin of C/EBP*β*. Then KDM6B promotes proliferation and metastasis in ESCC cells partly through activating the transcription of C/EBP*β* by demethylating the repressive H3K27me3 markers on the promoters (Fig. 6).

### 7 GSK-J4 inhibits proliferation, migration and invasion of ESCC cells

**Fig. 7:**
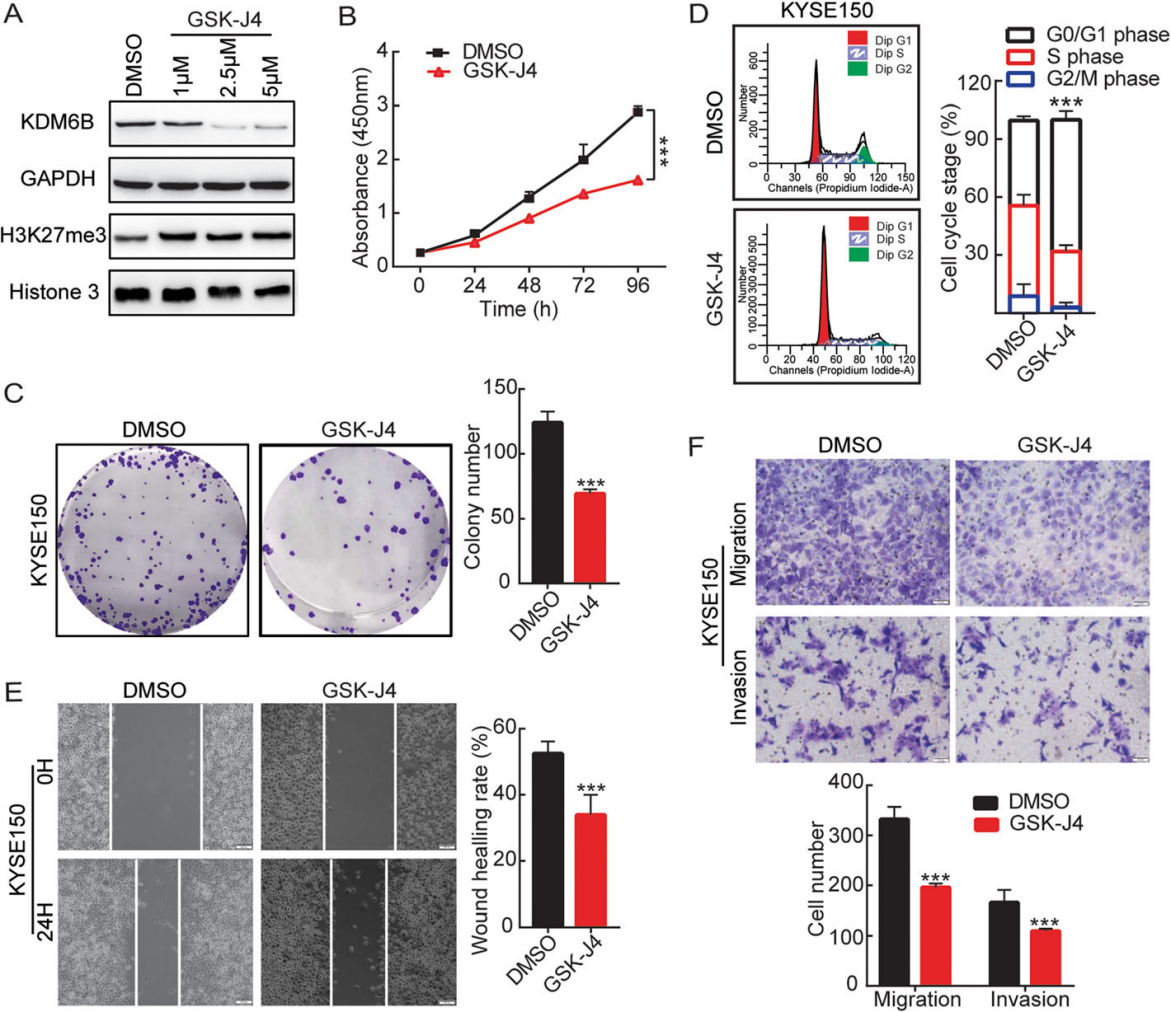
GSK-J4 inhibits proliferation, migration and invasion of ESCC cells. **a** KYSE150 cells were treated with the indicated concentrations of GSK-J4 for 24 h. western blots analysis the indicated antibodies. **b** The CCK-8 assay was used to determine cell proliferation of ESCC cells after treated with GSK-J4 for 24 h. **c** Representative images (left panel) and the statistical result (right panel) of cladogenesis abilities. **d** Representative images of cell cycle distribution (left panels) and statistical graphs of cell cycle changes (right panels). Cell scratch wound-healing assays **e**, Transwell migration and invasion experiment **f** detect the effect of GSK-J4 on migration and invasion ability KYSE150 cells. The bar graph shows the quantitative results of scratch healing and Transwell assay. Results are representative of three independent experiments and are expressed as the mean ± S.D. * p < 0.05, ** p < 0.01, *** p < 0.001

To further analyze the role of histone demethylase KDM6B inhibitor GSK-J4 on ESCC cells. KEYS150 cell was treated with the indicated concentrations of GSK-J4(1,2.5, 5 μM). The results showed that KDM6B expression was significantly down-regulated (Fig. 7a, Sup Fig. 3E), and H3K27me3 expression was significantly up-regulated. We chose 2.5 μM GSK-J4 for subsequent experiments. CCK8 assay showed that treatment of KYSE150 with GSK-J4 inhibits relative cell viability compared with DMSO control at 96 h (Fig. 7b), as expected, treat with GSK-J4 significantly decreased the clone formation ability of KYSE150 cells, and treat with GSK-J4 markedly induced G1/S cell cycle arrest in KYSE150 cells (Fig. 7c,d). These results show that GSK-J4 Induces a Block in the G1 Phase of the Cell Cycle, inhibits the proliferation and Clone formation of ESCC cells.

As previously stated, knockdown of KDM6B inhibits ESCC cell migration and invasion. We next explore whether the use of GSK-J4 to inhibit KDM6B has the same effect on ESCC. Figure 7e,f shows that when GSK-J4 treated KYSE150 cells, the migration and invasion rate was significantly reduced (*p* < 0.05). These results suggested that GSK-J4 can weaken ESCC cells’ migration and invasion.

## Discussion

In addition to DNA methylation, the importance of histone methylation in the transcriptional regulation is increasingly appreciated [31, 32]. H3K27me3/2 are repressive histone modifications important for gene silencing, and removal of such marks by KDM6B allows for activation of genes both during normal development and tissue differentiation as well as during carcinogenesis [20, 33]. Reports have shown that KDM6B has increased expression or abnormal activity in prostate cancer, breast cancer, kidney cancer, and other tumors, and plays a carcinogenic role [33–35]. The current role and mechanism of KDM6B in ESCC is not clear.

Li et al. found that KDM6B is overexpressed in ESCC and is associated with poor prognosis [36]. We have not reached such a conclusion, which may be due to the insufficient specimen. However, Our findings illustrate that the expression level of KDM6B was significantly increased in patients with lymph node metastasis. Previous studies have shown that KDM6B plays an important role in cell proliferation and metastasis. For example, KDM6B induces the expression of cervical cancer marker p16INK4A and promotes the proliferation of cancer cells [37]. In colon cancer, vitamin D up-regulates KDM6B and causes the expression of *ZEB1, ZEB2, SNAI* and other metastasis-related genes [38]. Our results indicate that KDM6B promotes the proliferation and migration of ESCC cells. However, it has also been reported that KDM6B appears as a suppressor gene in tumor [39, 40]. It can be seen that KDM6B currently plays a contradictory role in promoting or suppressing cancer. Consistent with this, another demethylase KDM6A mainly functions as a tumor suppressor gene, and its mutation has led to the occurrence of many tumors [41]. In recent years, it has also been reported that KDM6A can function as an oncogene in tumors. For example, in T-ALL driven by transcription factor TAL1, KDM6A acts as a co-activator of oncogene reprogramming [42]. Besides, EZH2 (Enhancer of Zeste Homology 2, Zeste homology enhancer 2), as the catalytic subunit of the PRC2 complex, can promote the methylation of H3K27, thereby epigenetically silencing the gene [43]. EZH2 is highly expressed in many tumors, and its inhibitor Tazemetostat has shown good safety and antitumor activity in refractory B-cell non-Hodgkin’s lymphoma and advanced solid tumors (including epithelioid sarcoma) [44]. EZH2 and KDM6A / B play the opposite role in catalyzing the methylation of H3K27, but they can also exhibit cancer-promoting activity. The above results indicate that it is far from sufficient to rely solely on the methylation of KDM6A / B and EZH2 on H3K27 to explain its function in tumors. The combination of histone modifications and other regulatory processes can affect overall biological outcomes, and the dysregulation of methylases and demethylases in tumors can also lead to different consequences. It depends on the primary tumor tissue, the presence of other mutations and other gene expression network activities. The mechanism is unclear.

we found that TNFA_SIGNALING_VIA_NFκB, INTERFERON_ GAMMA_RESPONSE, P53, and IL6_JAK_STAT3_SIGNALING pathway were significantly suppressed in KDM6B knockdown KYSE150 as compared with Ctrl cells, and those signaling pathways were known to be closely associated with oncogenesis. KDM6B, JUNB, and C/EBP*β* are key regulatory gene for the TNFA_SIGNALING_VIA_NFκB pathway. Yu et al. pointed out that genes such as JUNB, C/EBP*β*, and CCNG2 are the target genes of KDM6B. Importantly, C/EBP*β* is one of the major transcription factors that recruit KDM6B to the target gene promoter. Almost eliminated the occupancy rate of KDM6B on these target gene promoters [44]. Our results preliminarily verified that KDM6B regulates the expression of C/EBP*β* by demethylating H3K27me3 and promotes the progress of ESCC. The above research enlightens us that some proteins play an important role in the process of KDM6B promoting tumor progression, so how these proteins interact with KDM6B is the focus of our further research.

At present, individualized tumor treatment driven by biomarkers has attracted great attention. More and more studies have shown that KDM6B can be used as a biomarker and therapeutic target for tumor diagnosis and prognosis. Our research also found that the KDM6B inhibitor GSK-J4 can inhibit the proliferation and migration of ESCC cells. However, whether KDM6B plays a carcinogenic or anticancer role in certain tumors may depend on the specific tissue and cell type. Therefore, the use of KDM6B as a new treatment requires a broader and deeper understanding of its biological function and molecular mechanism.

## Conclusion

In conclusion, our results suggest that KDM6B was highly overexpressed in metastatic ESCC tissues, and KDM6B promotes ESCC progression by increasing the transcriptional activity of C/EBP*β* depending on its H3K27 demethylase activity. KDM6B demethylase inhibitor, GSK-J4, acts to restore K27 methylation in ESCC cells, while demonstrating potent anti-tumor activity. Thus, we identify KDM6B as an oncogenic factor of ESCC and suggest KDM6B might be a potential molecular target for therapeutic approaches for ESCC.

## Supplementary Information

**Additional file 1.**

**Additional file 2.**

## Data Availability

The datasets used and/or analyzed during this study are available from the corresponding author on reasonable request. Information on RNA-seq and ChIP-seq are available in https://www.ncbi.nlm.nih.gov/sra/PRJNA691394, the submission ID are SUB8898024 and SUB8899697, respectively.

## Abbreviations

ESCC: Esophageal squamous cell carcinomas
KDM6B: Lysine*-*specific demethylase 6B
H3K27me3: Trimethylation of histone H3 lysine 27
RNA-seq: RNA-sequencing
ChIP-seq: Chromatin immunoprecipitation–sequencing
GSEA: Gene Set Enrichment Analysis
