# Correction to: KDM6B promotes ESCC cell proliferation and metastasis by facilitating C/EBP*β* transcription

**DOI:** 10.1186/s12885-021-08625-7

**Published:** 2021-08-05

**Authors:** Mei Qin, Fei Han, Jian Wu, Feng-xia Gao, Yuan Li, De-xin Yan, Xue-mei He, Yang Long, Xiao-ping Tang, De-lian Ren, Yan Gao, Tian-yang Dai

**Affiliations:** 1 Department of Immunology, Basic Medicine College, South West Medical University, Luzhou, Sichuan China; 2 Department of Thoracic Surgery, The Affiliated Hospital of Southwest, Medical University, Luzhou, Sichuan China; 3 Experimental Medicine Center, The Affiliated Hospital of Southwest Medical University, Luzhou, Sichuan China

**Correction to: BMC Cancer 21, 559 (2021)**

**https://doi.org/10.1186/s12885-021-08282-w**

Following publication of the original article [1], the authors identified an error in the author affiliation of Tian-yang Dai. The affiliation, should be:

2 Department of Thoracic Surgery, The Affiliated Hospital of Southwest, Medical University, Sichuan, Luzhou, China.

The author group has been updated above and the original article [1] has been corrected.
