# Supplementary material for: KDM6B promotes ESCC cell proliferation and metastasis by facilitating C/EBPβ transcription
Source: BMC Cancer. 2021 May 17;21:559. doi: 10.1186/s12885-021-08282-w (PMC8130268; doi:10.1186/s12885-021-08282-w)
Supplement: Supplementary file 1 — Additional file 1. [file 12885_2021_8282_MOESM1_ESM.docx]

## KDM6B promotes ESCC cell proliferation and metastasis by facilitating C/EBP*β* transcription

Mei Qin^2+^, Fei Han^1+^, Jian Wu^1^, Feng-xia Gao^2^, Yuan Li^1^, De-Xin Yan^1^, Xue-mei He^3^, Yang Long ^3^，Xiao-ping Tang^3^，De-lian Ren^2^, Yan Gao^2*^，and Tian-yang Dai^1*^

### Supplementary Figure 1 The overexpression of KDM6B promotes the proliferation and Metastasis of ESCC cells. (A) Western blotting and RT-qPCR were performed to measure the KDM6B protein and mRNA expression level changes following KDM6B overexpression. (B) The CCK-8 assay was used to determine cell viability after overexpressing KDM6B every 24 h for 4 days. (C) ESCC cells were seeded at a density of 500 cells/well for clone formation assays. Representative crystal violet staining of clone formation (upper panels) and statistical graphs of clone numbers (lower panels). (D) Cell cycle distribution was analyzed following the overexpressing of KDM6B in Eca9706 and TE11. Representative images of cell cycle distribution (left panels) and statistical graphs of cell cycle changes (right panels).. (E) Cell migration was determined using the wound healing migration assay (magnification, ×100) Representative images of Eca9706 and TE11 HA-KDM6B transfected at 0 and 24h of wound healing assay. Image j was used to analyze the results of wound scratch images. A bar graph shows the quantitative results of scratch healing. (F) Cell migration or invasion was determined using Transwell migration or invasion assay. Representative images of crystal violet stained for HA-KDM6B transfected of Eca9706 and TE11 by transwell migration or invasion assays. A statistical graph shows Cell number(lower panels). Results are representative of three independent experiments and are expressed as the mean±S.D. * *p* <0.05, ** *p* <0.01, *** *p* <0.001.

### Supplementary Figure 2 Interference with C/EBP*β* inhibits the proliferation, migration and invasion of ESCC cells. A RT-qPCR was performed to measure the C/EBP*β* mRNA expression level changes following C/EBP*β* interference. B The CCK-8 assay was used to determine cell proliferation of ESCC cells after transfection with C/EBP*β* every 24 h for 4 days. C KYSE150 cells were seeded at a density of 500 cells/well for clone formation assays. Representative crystal violet staining of clone formation (left panels) and statistical graphs of Clone numbers (right panels). (D) Cell cycle distribution was analyzed following the silencing of C/EBP*β* in KYSE150. Representative images of cell cycle distribution (left panels) and statistical graphs of cell cycle changes (right panels). (E) Cell migration was determined using the wound healing migration assay (magnification, ×100) Representative images of KYSE150 lentivirus stable transfected at 0 and 24h of wound healing assay. Image j was used to analyze the results of wound scratch images. A bar graph shows the quantitative results of scratch healing. (F) Cell migration or invasion was determined using Transwell migration or invasion assay. A representative images of crystal violet stained for lentivirus stable transfected of KYSE150 by transwell migration or invasion assays. A statistical graph shows Cell number(right panels). Results are representative of three independent experiments and are expressed as the mean±S.D. * *p* <0.05, ** *p* <0.01, *** *p* <0.001.

### Supplementary Figure 3  Relative gray scale of protein expression determined following WB analysis. A, B, C, D and E are the ratio of gray value of target protein and gray value of internal reference in figure 1E, figure 2A, figure 4H, figure 4I, and figure 6A respectively. GAPDH and Histone3 were used as the internal reference. Results are representative of three independent experiments and are expressed as the mean±S.D. * *p* <0.05, ** *p* <0.01, *** *p* <0.001.


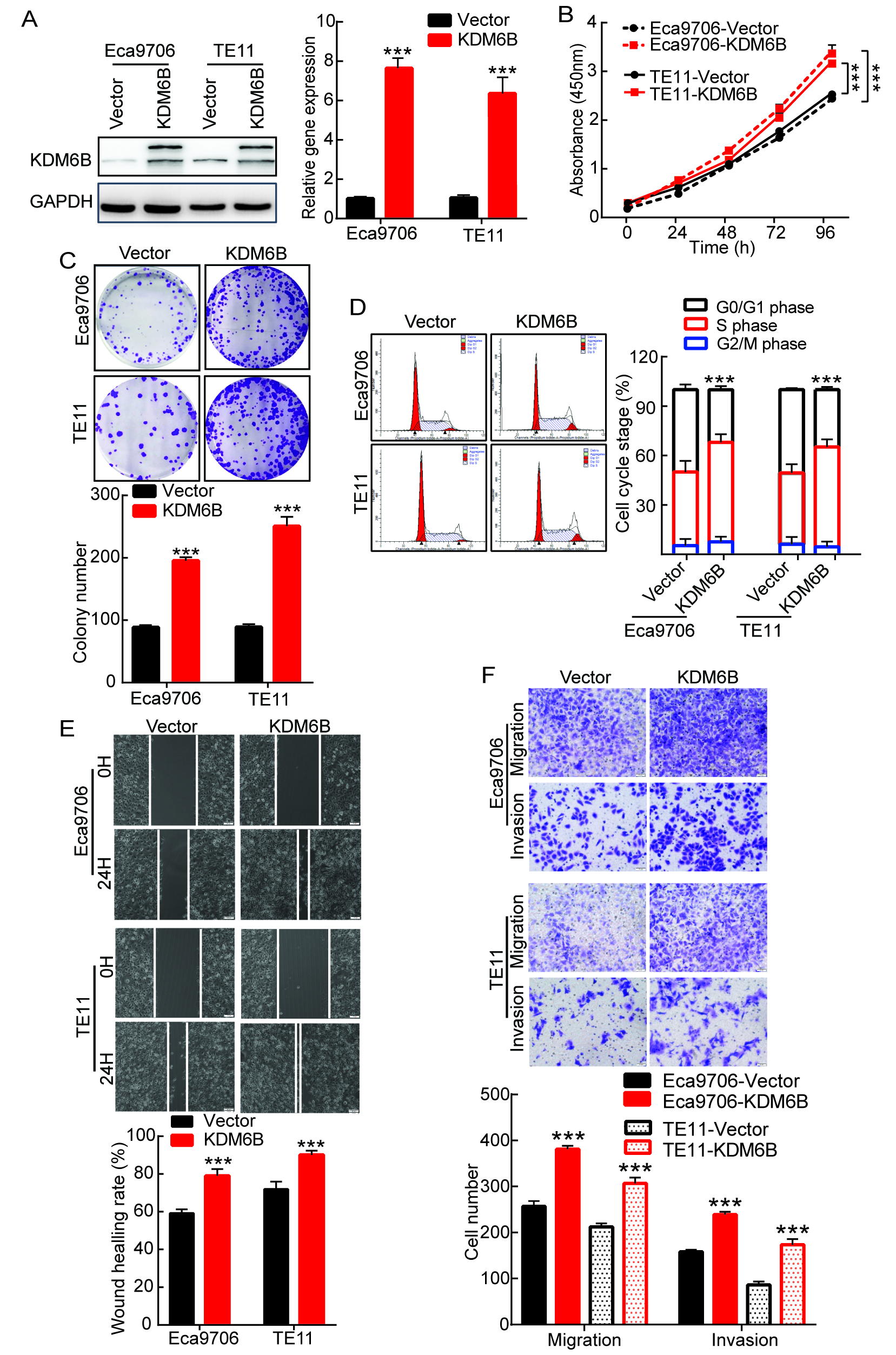
Supplementary Figure 1­


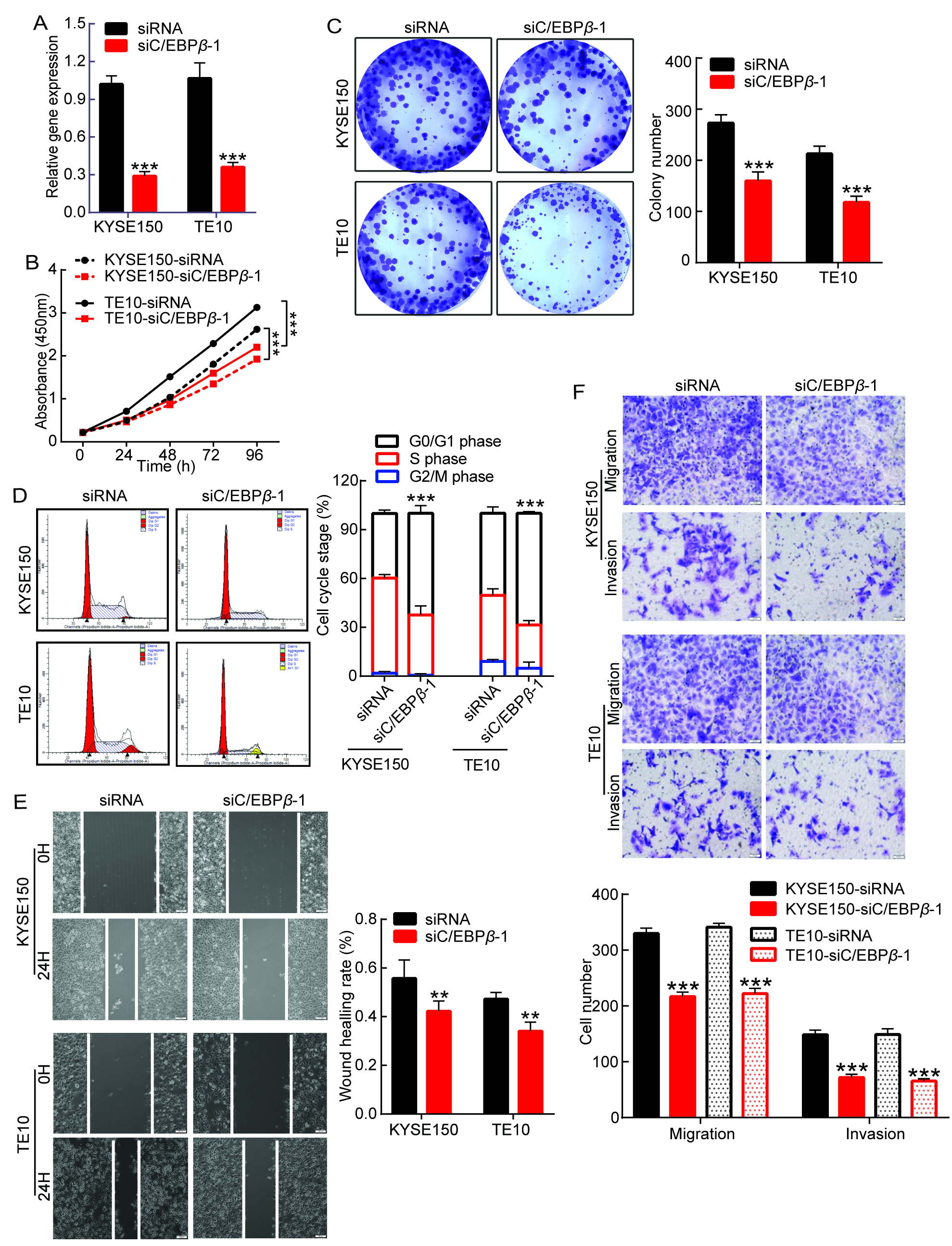
Supplementary Figure 2


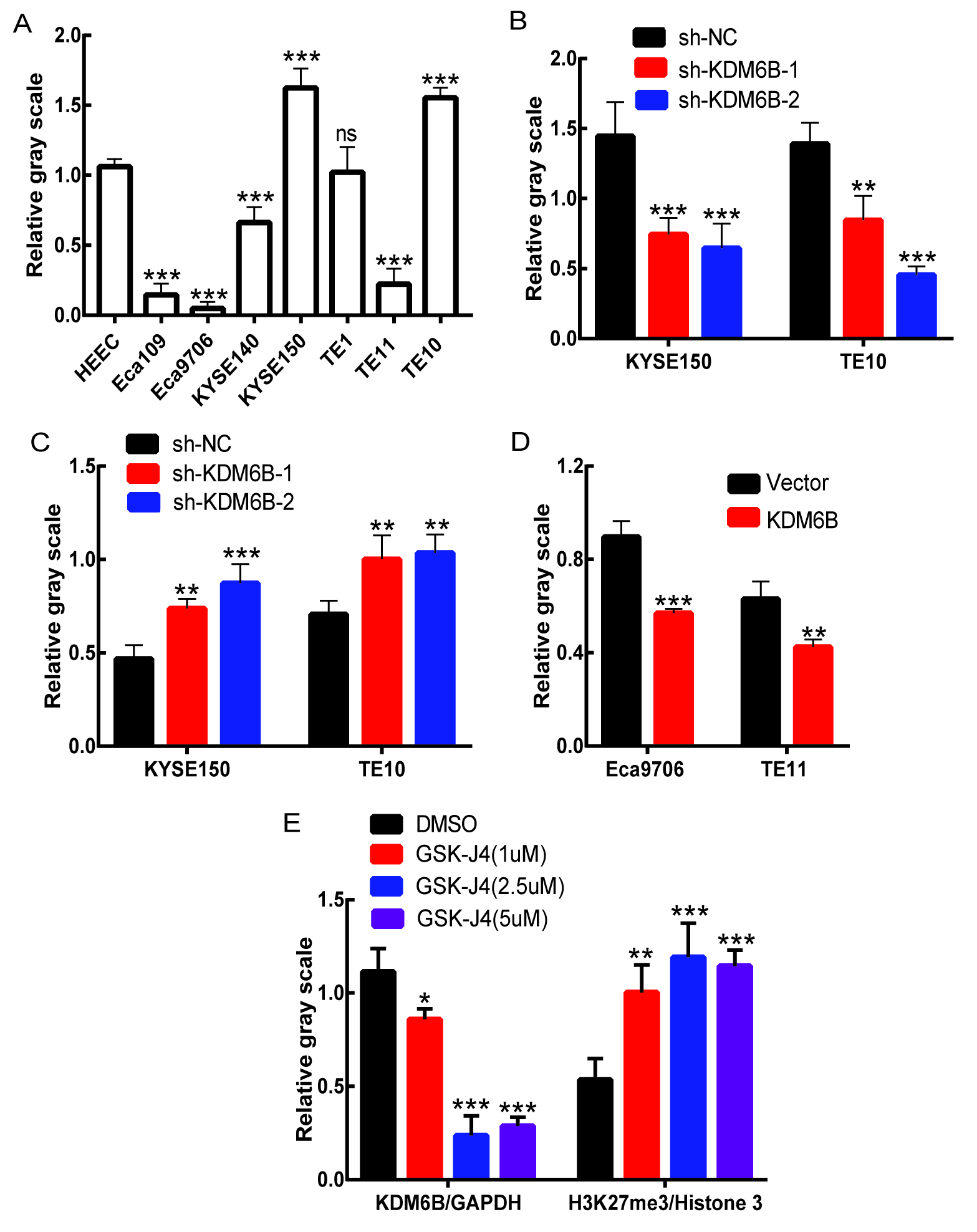
Supplementary Figure 3
