# Supplementary material for: KDM6B promotes ESCC cell proliferation and metastasis by facilitating C/EBPβ transcription
Source: BMC Cancer. 2021 May 17;21:559. doi: 10.1186/s12885-021-08282-w (PMC8130268; doi:10.1186/s12885-021-08282-w)
Supplement: Supplementary file 2 — Additional file 2. [file 12885_2021_8282_MOESM2_ESM.docx]

­­

| FIG1E(KDM6B) | 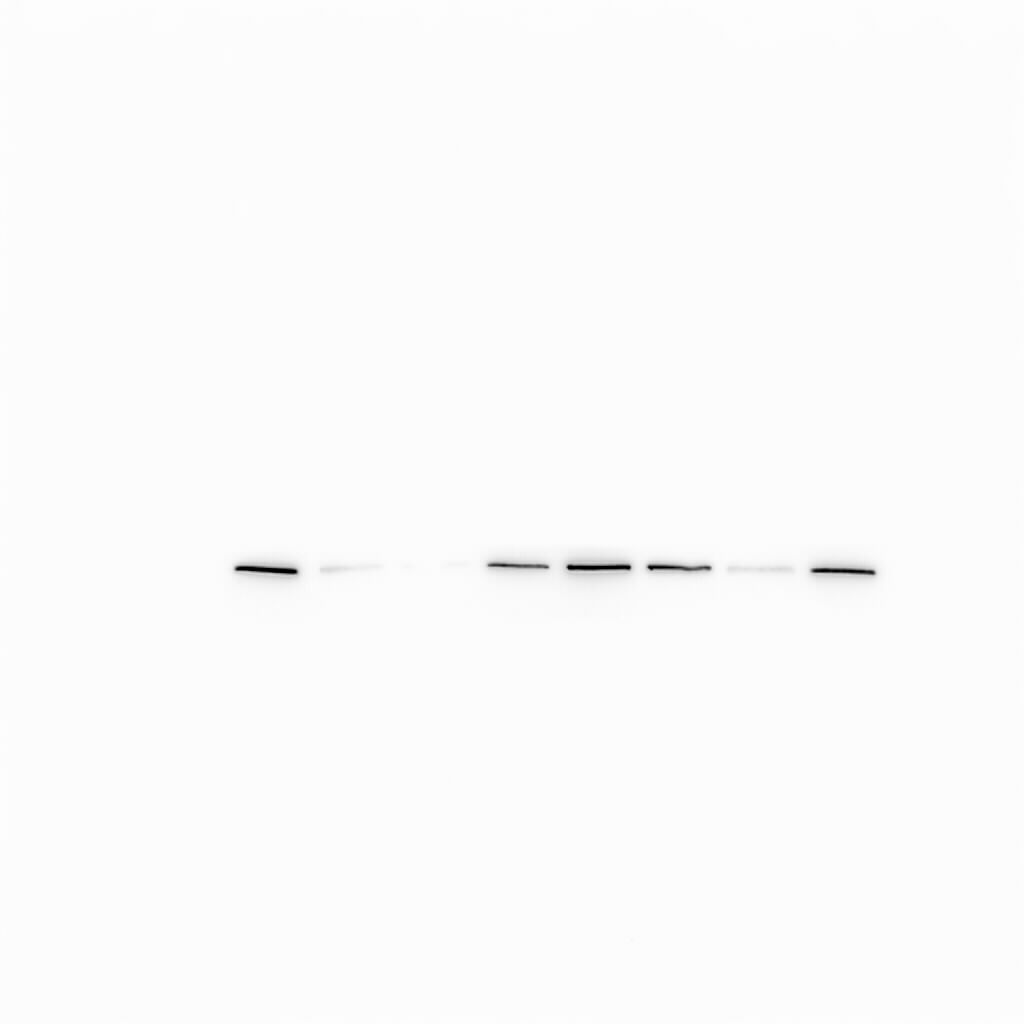 |
| --- | --- |
| FIG1E(GAPDH) | 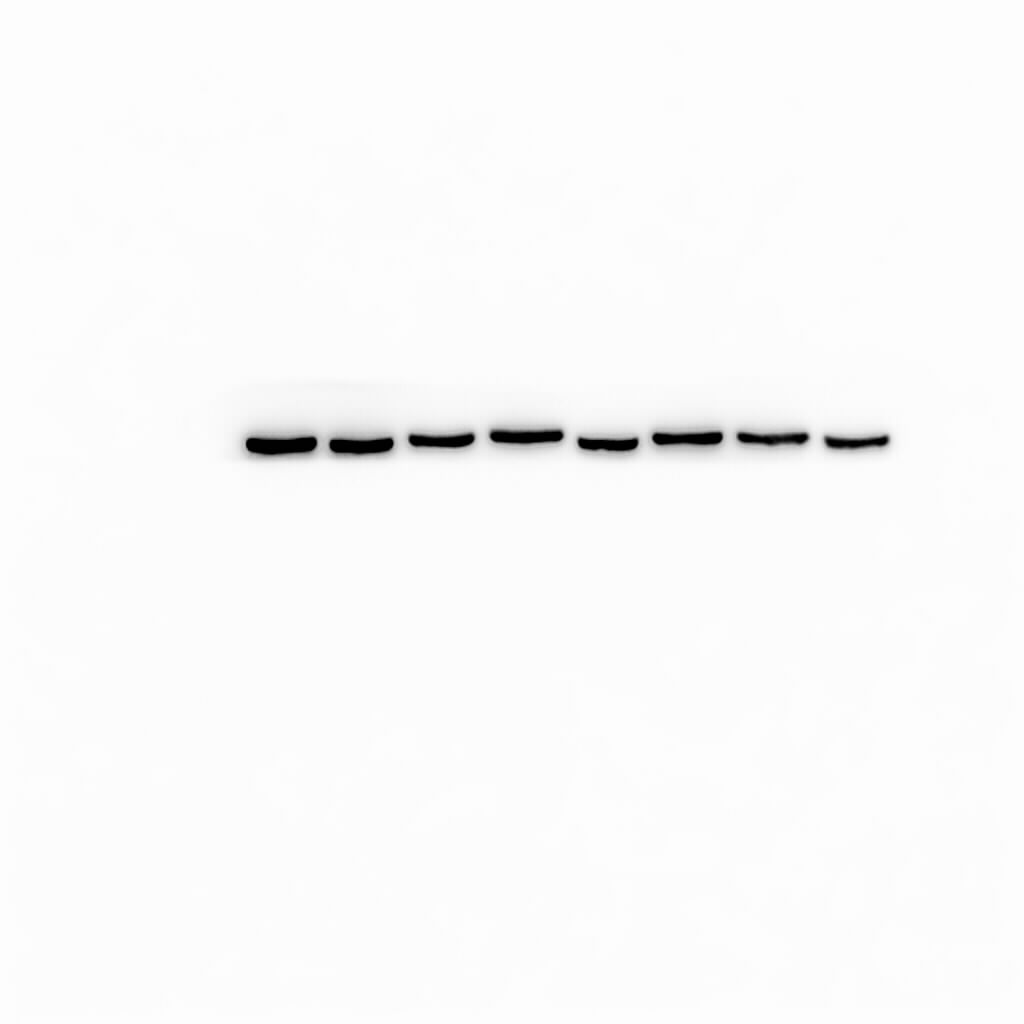 |
|  | the expression of KDM6B among HEEC cells and ESCC cell lines by Western blot (Fig. 1E). Compared with HEEC, the expression of KEM6B was significantly higher for KYSE150, TE10, and significantly lower for Eca9706, TE11. |
| FIG2A(KDM6B) | 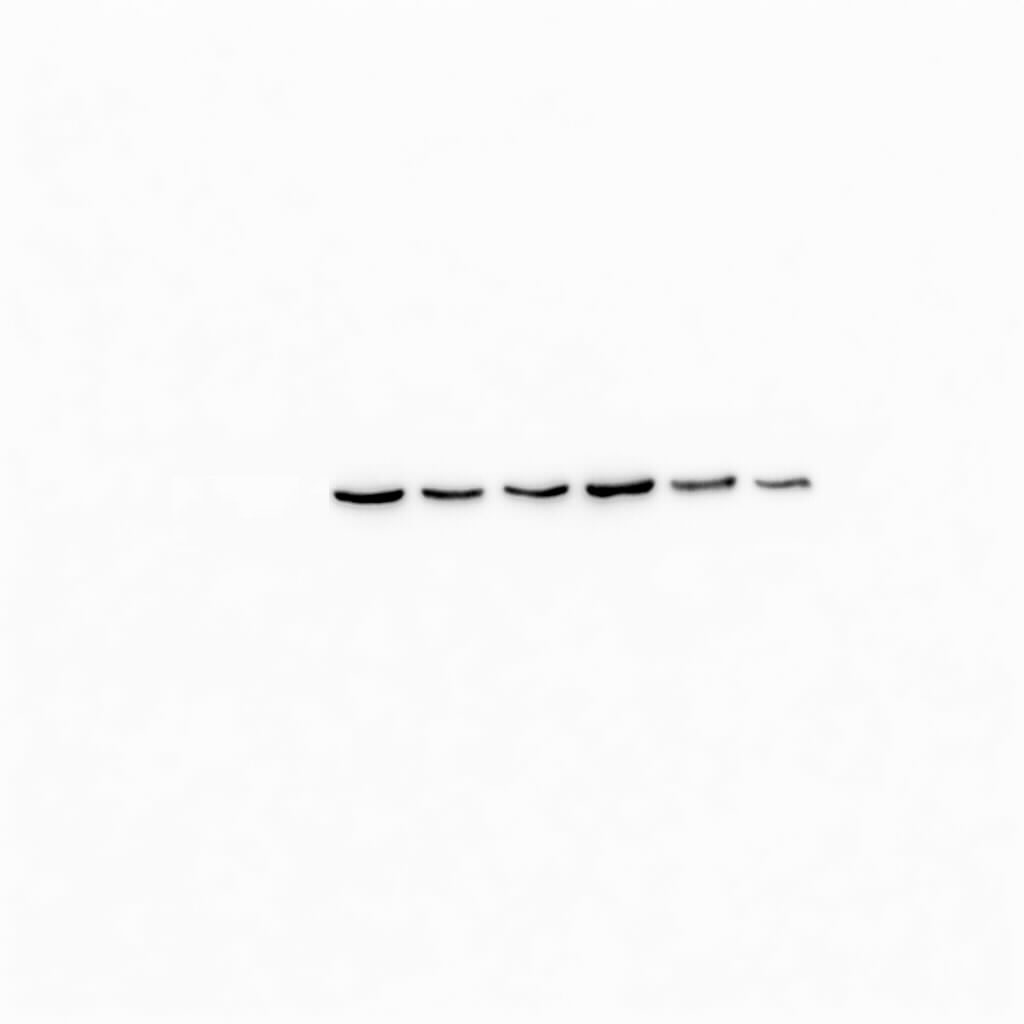 |
| FIG2A(GAPDH) |  |
|  | We use Western blot to determine the knockdown efficiency, compared with the sh-NC group, the protein expressions of KDM6B were significantly inhibited in cells transfected with sh-KDM6B. |
| FIG4H(H3K27me3) |  |
| FIG4H（Histone3） |  |
| FIG4I(H3K27me3) | 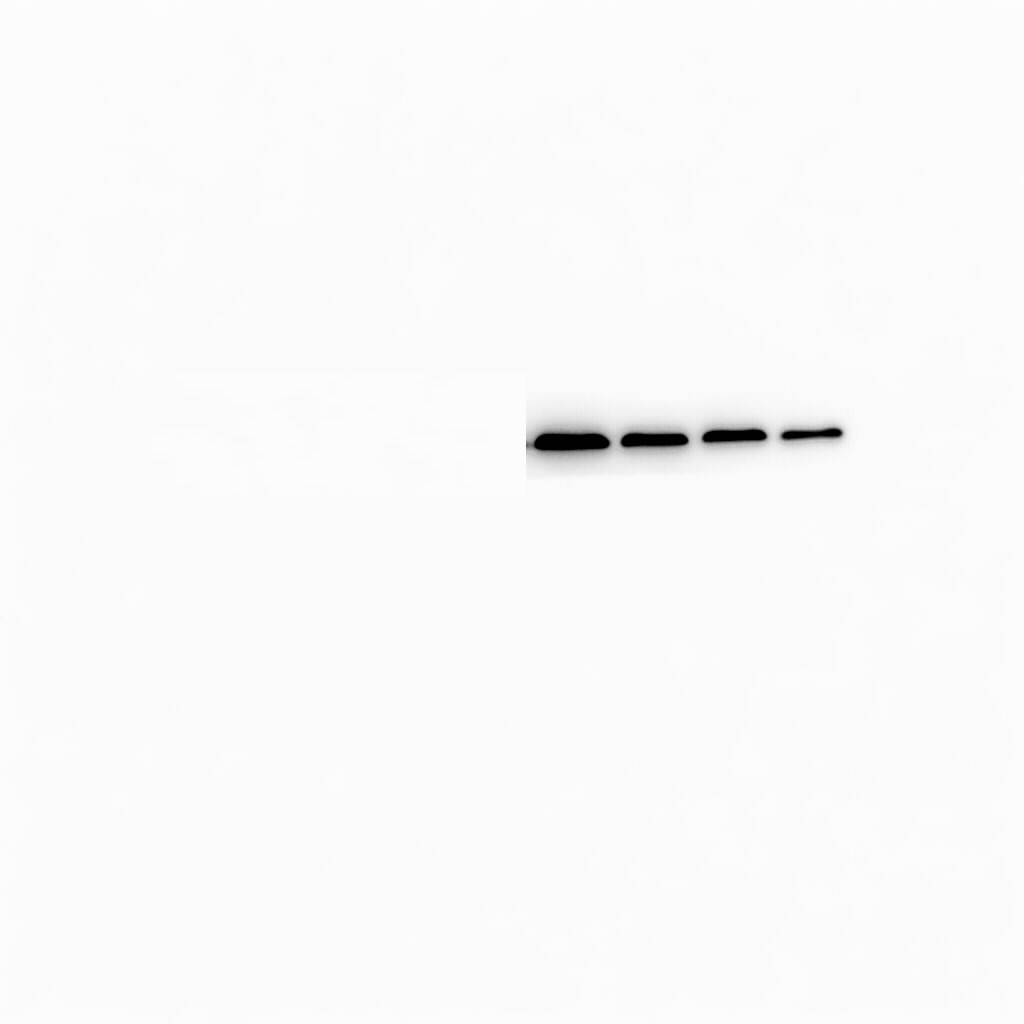 |
| FIG4I（Histone3） |  |
|  | (H) Western blot was used to detect the expression of H3K27me3 in KYSE150 and TE10 cells after the KDM6B knockdown. (I) Western blot detected the expression of H3K27me3 in Eca9706, TE11 after over-expressing KDM6B for 72 h.  we found that after interfering with KDM6B, the expression of H3K27me3 was enhanced, and after overexpressing of KDM6B, the expression of H3K27me3 was significantly reduced. |
| FIG6A(KDM6B) | 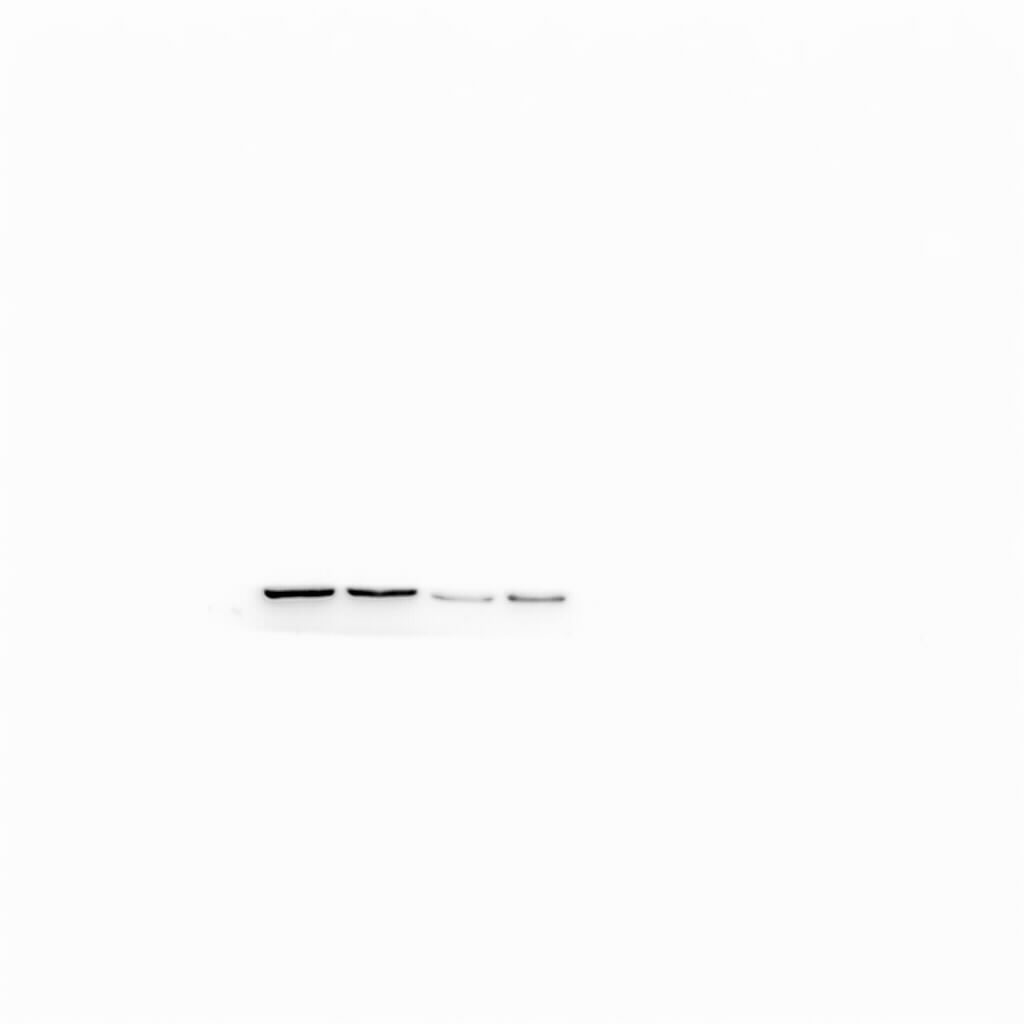 |
| FIG6A(GAPDH) | 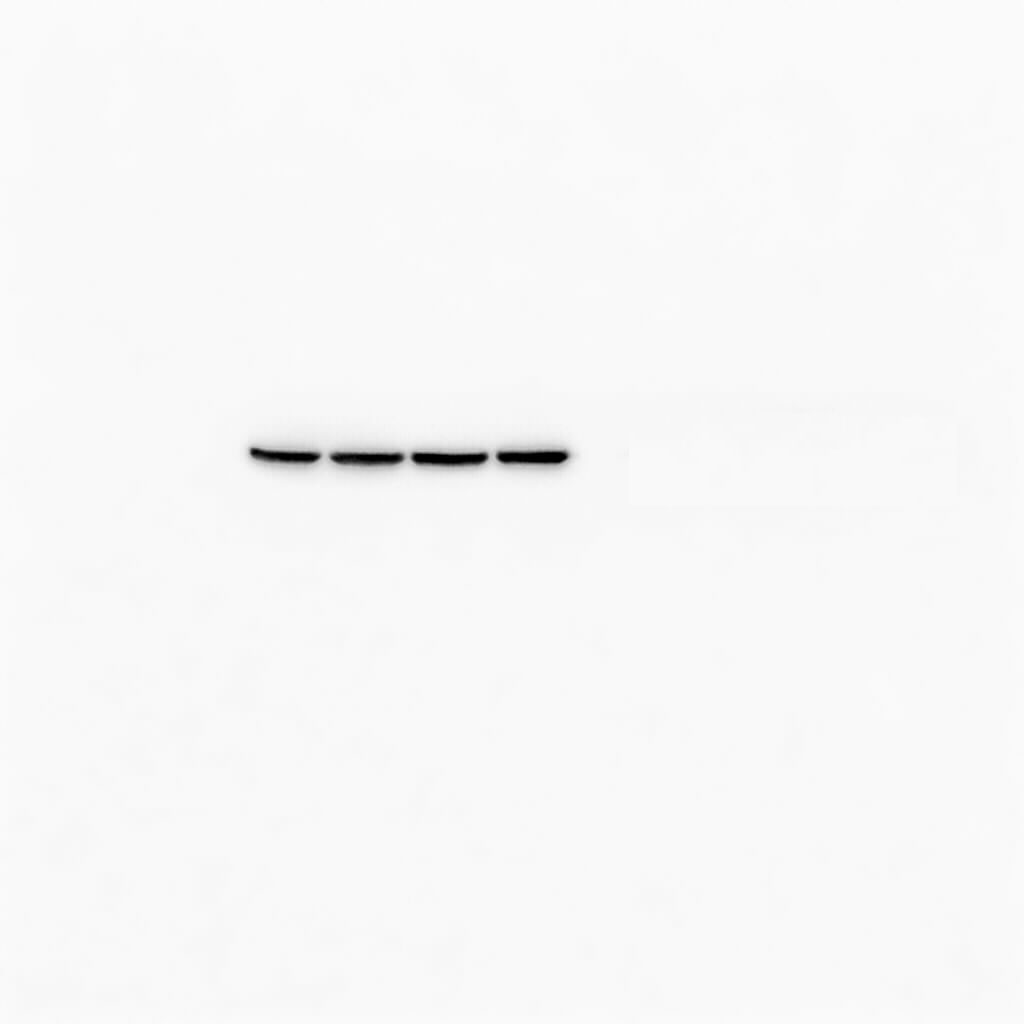 |
| FIG6A(H3K27me3) |  |
| FIG6A（Histone3） |  |
|  | KYSE150 cells were treated with the indicated concentrations of GSK-J4 (1,2.5, 5µM) for 24 h. western blots analysis the indicated antibodies. The results showed that KDM6B expression was significantly down-regulated(Fig. 6A), and H3K27me3 expression was significantly up-regulated after the cells were treated with GSK-J4. |
| Sup.FIG1A(KDM6B) | ­­­­­ |
| Sup.FIG1A(GAPDH) | 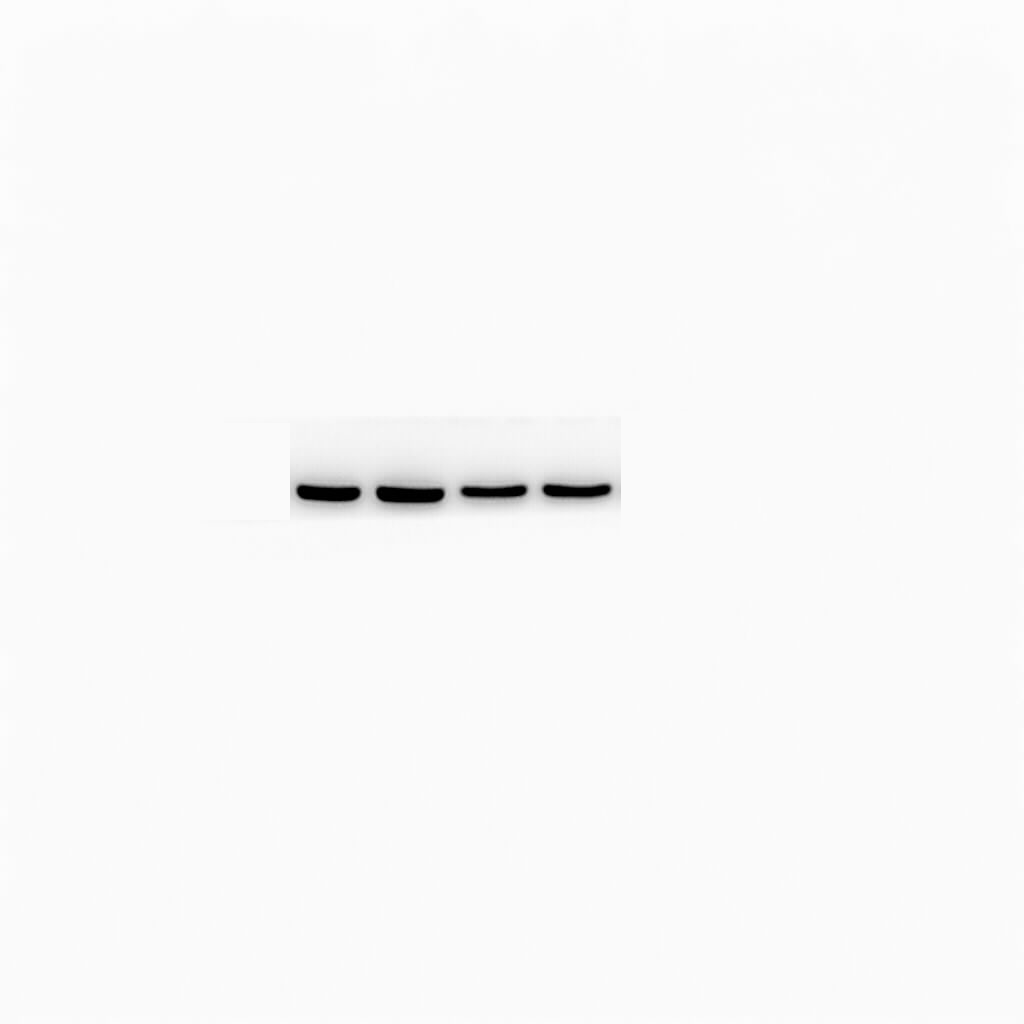 |
|  | Western blotting was performed to measure the KDM6B protein level changes following KDM6B overexpression in ECA9706 and TE11. |
